# Adipocyte-derived IL6 and triple-negative breast cancer cell-derived CXCL1 co-activate STAT3/NF-κB pathway to mediate the crosstalk between adipocytes and triple-negative breast cancer cells

**DOI:** 10.1038/s41420-025-02713-4

**Published:** 2025-08-21

**Authors:** Guo-Tian Ruan, Li-Chen Zhu, Hai-Lun Xie, He-Yang Zhang, Meng-Meng Song, Li Deng, Han-Ping Shi

**Affiliations:** 1 Department of General Surgery, Beijing Friendship Hospital, Capital Medical University, Beijing, China; 2 Department of Gastrointestinal Surgery/Department of Clinical Nutrition, Beijing Shijitan Hospital, Capital Medical University, Beijing, China; 3 National Clinical Research Center for Geriatric Diseases, Xuanwu Hospital, Capital Medical University, Beijing, China; 4 Key Laboratory of Cancer FSMP for State Market Regulation, Beijing, China; 5 Laboratory for Clinical Medicine, Capital Medical University, Beijing, China

**Keywords:** Cancer microenvironment, Breast cancer, Breast cancer, Prognostic markers

## Abstract

Triple-negative breast cancer (TNBC) is correlated to a poor prognosis, especially in the context of obesity. The interaction between adipocytes and TNBC cellsplay a key role in the progression of TNBC. This study aims to investigate the mechanisms underlying the cross-talk and progression between adipocytes and TNBC cells. We established a co-culture model involving mature adipose cells (hADSC and 3T3-L1) and TNBC cells. Cell invasion abilities were assessed using wound healing and Transwell assays. Gene and protein expression levels were examined using RT-PCR, western blotting, and immunostaining. Adipocytokine and chemokine levels were measured using ELISA. Additionally, we developed a fat mouse model induced by a high-fat diet and a tumor-bearing model of TNBC cells in vivo. The results indicated a significant enhancement in the invasion abilities of TNBC cells after co-culture. Mature adipose tissue co-cultured with TNBC cells increased the expression and secretion of C-X-C motif chemokine ligand 1 (CXCL1) and upregulated matrix metalloproteinase 7 (MMP7) and MMP9 in TNBC cells by activating the signal transducer and activator of transcription 3 (STAT3) /nuclear factor-κB p65 (NF-κB p65) pathway. Additionally, co-culture activated the STAT3/NF-κB p65 pathway, increasing the expression and secretion of IL6 in adipocytes. Based on the mouse obesity model, our experiments on orthotopic breast fat pad xenoimplantation showed consistent results in vivo. Our findings suggest a cross-talk between TNBC cells and adipocytes, activating the STAT/NF-κB p65 pathway through the production and secretion of CXCL1 and IL6, respectively, thereby promoting TNBC progression. These results propose a potential strategy for developing individualized treatments for patients with TNBC in clinical practice.

## Introduction

Breast cancer (BC) holds the top spot globally in both new cases and female tumor-related deaths [1]. Triple-negative breast cancer (TNBC) is a particular clinical subtype characterized by the absence of estrogen receptor (ER), progesterone receptor (PR), and human epidermal growth factor receptor-2 (HER2) proteins receptors, making up ~15% of BC [2]. Patients with TNBC face challenges such as an invasive phenotype, poor prognosis, heightened metastatic potential, lack of specificity, and absence of therapeutic targets [3, 4]. Currently, the main treatment for TNBC is chemotherapy; however, it has less than optimal outcomes.

Obesity is rapidly becoming a global epidemic [5]. It is the most prevalent metabolic disease worldwide, and its incidence is rising rapidly [6]. During and after treatment, about 35% of patients with BC reported a weight gain ranging from 1.4 kg to 5.0 kg [7–9]. BC often manifests in or around adipose tissue, indicating that biological changes in fat actively promote localized BC development with an elevated body mass index (BMI) [10, 11]. Individuals who are obese exhibit a significantly higher mortality rate from breast cancer compared to people who are non-obese [12]. Reports indicate that women with a BMI > 40 kg/m^2^ suffer a higher risk of death (relative risk = 2.12) [13]. Furthermore, obesity correlates with positive lymph nodes, larger tumors size, shorter disease-free intervals, overall survival [14–16], and TNBC [15].

Our previous study found that systemic inflammation is correlated to higher BMI and poor survival outcome in patients with BC [17]. Obesity causes systemic metabolic disorders, resulting in dyslipidemia, hypercholesterolemia, insulin resistance, altered hormone levels, and shifts in inflammatory baselines [18]. Inflammation or inflammatory disorders induced by obesity are the main features of adipose tissue dysfunction [19]. Adipose tissue not only serves as a storage site for excess energy in the form of triglycerides but is also recognized as an active and complex endocrine organ that secretes various polypeptides known as adipose cytokines [20]. In obese individuals, the production and expression of inflammatory adipose cytokines, such as interleukin-6 (IL6) and tumor necrosis factor-alpha [21]. Obesity-associated adipose tissue often displays unhealthy characteristics, including decreased blood vessel density, adipocyte hypertrophy, and inflammation [22, 23]. Alterations in the inflammation of breast adipose tissue can impact early BC [24]. The tumor microenvironment (TME) is a heterogeneous ecosystem comprising infiltrating immune cells, mesenchymal supporting cells, and matrix components that collectively promote tumor progression. Within the BC microenvironment, adipocytes are the main cellular constituents. Recent evidence underscores the role of adipocytes in promoting tumor progression through the mutual communication between tumor cells and adipocytes [25, 26]. While numerous studies have explored the impact of adipocytes on tumor cells, the mechanism of action of tumor cells on adipocytes has rarely been reported, and the uncertainty of the interaction between them still needs to be explored. Close interaction between BC cells and adipocytes results in cancer-associated adipocytes (CAAs) exhibiting distinctive phenotypic, gene expression, and secretory characteristics [25, 27]. Co-culturing tumor cells with adipocytes enhances the invasive potential of both cells, accompanied by metabolic adaptations that fuel tumor progression. Obesity may contribute to the generation of adipocytes that mimic the CAAs phenotype by secreting pro-inflammatory cytokines, growth factors, and extracellular matrix proteins, thereby providing a nutrient-rich environment that accelerates the growth of early and late tumors. Consequently, this poses an increased risk of BC mortality or tumor progression [28].

The high-fat TME observed in TNBC is associated with cell proliferation, migration, angiogenesis, inhibition of apoptosis, alterations in immune response, and development of drug resistance. When there is a high-fat microenvironment in patients with TNBC, more attention should be paid to its accumulation and superposition. Thus, this study aims to investigate the cross-talk mechanism between TNBC cells and adipocytes and the potential prognostic impact of the high-fat microenvironment on TNBC cells. This study will deepen our understanding of the impact of high-fat microenvironment on the progression of TNBC and provide a potential way for more effective clinical intervention and improving the prognosis of patients.

## Results

### Co-culture with adipocytes can promote the migration and invasion of TNBC cells

**Fig. 1:**
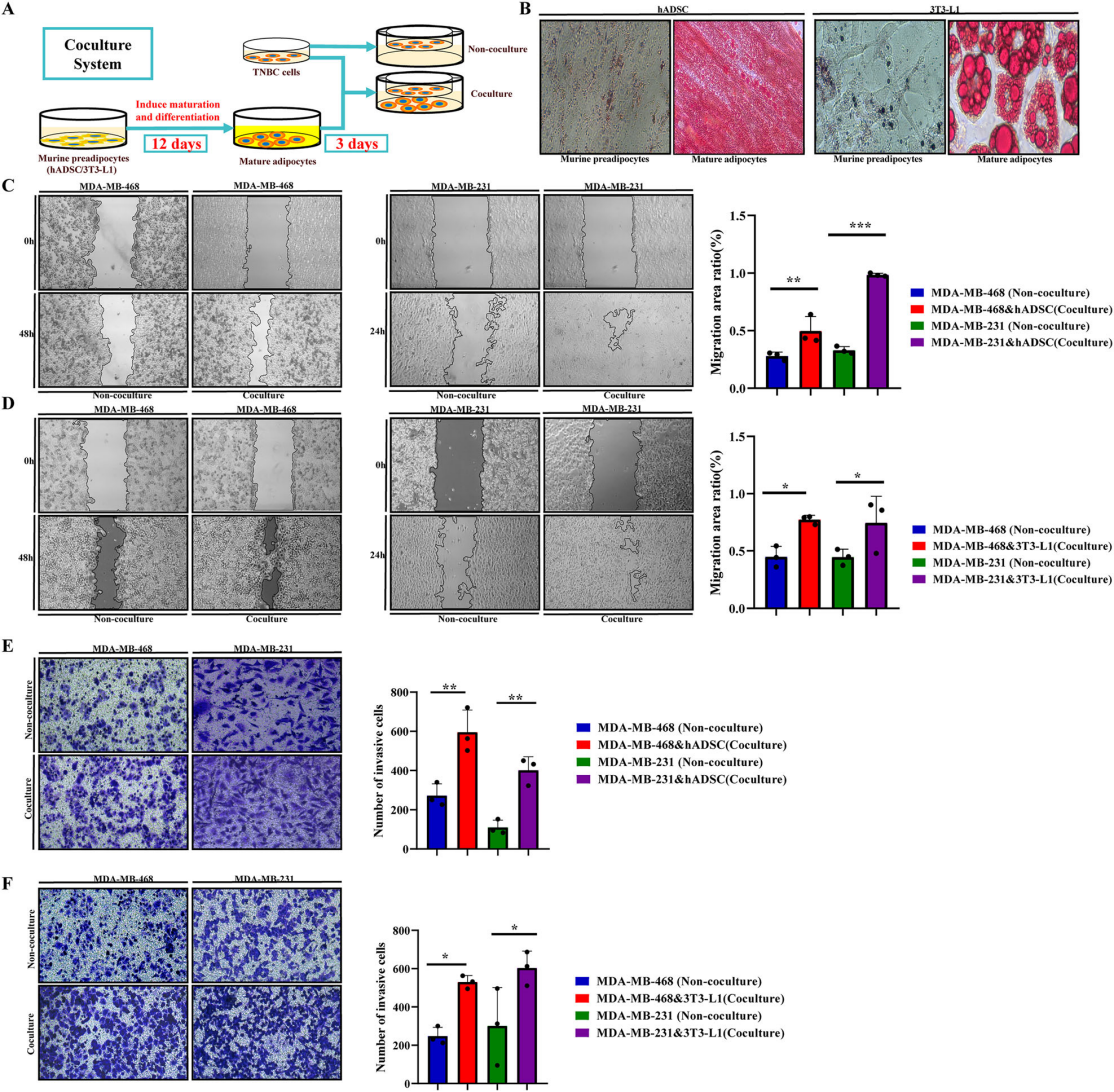
Co-culture with adipocytes promotes the migration and invasion of TNBC cells. **A** The schematic diagram of co-culture model construction. Adipocytes hADSC and 3T3-L1 cells were induced to differentiate into mature adipocytes after 12 days of maturation, adipocytes in the lower chamber and TNBC cells in the compartment were co-cultured for 3 days; **B** Oil red O staining of adipocytes. Mature adipocytes secrete large amounts of lipid droplets; **C**, **D** Co-culture with adipocytes promotes the migration ability of TNBC cells. MDA-MB-468 or MDA-MB-231 cells were co-cultured with adipocytes or single inoculation for 3 days, and the migration experiment was carried out. **C** Co-culture with human hADSC cells. **D** Co-culture with mouse 3T3-L1 cells; **E**, **F** Co-culture with adipocytes promotes the invasion ability of TNBC cells. MDA-MB-468 or MDA-MB-231 cells were co-cultured with adipocytes or single inoculation for 3 days, and the invasion experiment was carried out. **E** Co-culture with human hADSC cells. **F** Co-culture with mouse 3T3-L1 cells. Error bars represent means ± SD. *P < 0.05, **P < 0.01, ***P < 0.001.

We constructed a co-culture system of adipocytes and TNBC cells, enabling the intercommunication of cytokines secreted by the cells through the pores of the chambers (Fig. 1A). Mature adipocytes can secrete lipid droplets that can be visualized by Oil Red O staining (Fig. 1B). In this study, we used two different species of adipocytes for double analysis: human hADSC and mouse 3T3-L1 cells. The invasion abilities of MDA-MB-468 and MDA-MB-231 cells co-cultured with human hADSC and mouse 3T3-L1 cells were stronger than those of TNBC cells cultured alone, indicating that the adipocytes can promote the migration and invasion abilities of TNBC cells (*P* < 0.05) (Fig. 1C–F).

### Chemokines play important roles in the adipocyte-TNBC cell interaction

**Fig. 2:**
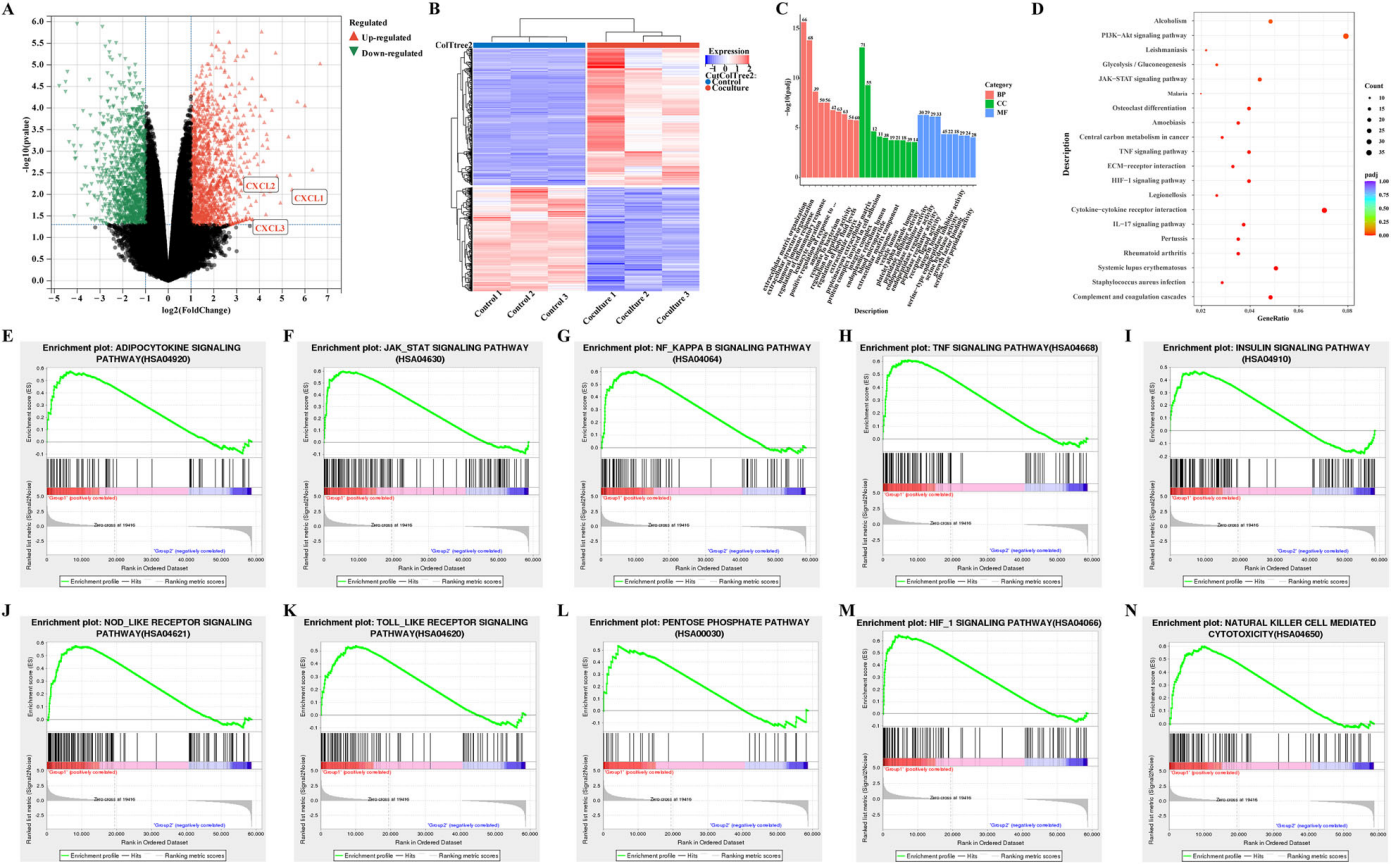
Transcriptional sequence analysis of MDA-MB-468 cells co-cultured with or without hADSC cells. MDA-MB-468 of TNBC cells were co-cultured with hADSC cells or cultured separately. After 3 days, the cells were collected for transcriptional sequencing. **A** Differential gene volcano plot, red indicates differentially expressed upregulated genes, green represents differentially downregulated genes; **B** Differential gene heat map, red indicates differentially expressed upregulated genes, blue represents differentially downregulated genes. *N* = 3 co-expressed MDA-MB-468 cells and *n* = 3 single cultured MDA-MB-468 cells; **C**, **D** GO terms bio-enrichment analysis and KEGG pathway analysis of differentially expressed genes. **C** GO terminology biological enrichment analysis; **D** Bubble diagram of KEGG; **E**–**N** Enrichment analysis of GSEA pathway of differentially expressed genes.

We explored the potential mechanism by which adipocytes promote the migration and invasion abilities of TNBC cells. There is an interaction between adipocytes and TNBC cells. We analyzed the transcriptional sequences of MDA-MB-468 cells co-cultured with human adipose-derived stem cell (hADSC) and of MDA-MB-468 cells cultured alone. The analysis revealed 778 upregulated and 592 downregulated genes in the co-cultured MDA-MB-468 cells (Fig. 2A, B). Enrichment and functional analysis of the differentially expressed genes indicated the involvement of co-cultured MDA-MB-468 cells in biological processes related to extracellular matrix management and inflammatory responses. Further analysis of the enrichment gene mechanism suggested that the co-cultured MDA-MB-468 cells may be involved in potential biological pathways and mechanisms such as “PI3K-Akt signaling pathway”, “JAK-STAT signaling pathway,” “TNF signaling pathway,” “ECM-receptor interaction,” “HIF-1 signaling pathway”, “Cytokine-cytokine receptor interaction,” and “IL-17 signaling pathway” (Fig. 2C, D). Gene Set Enrichment Analysis (GSEA) revealed that upregulated genes in MDA-MB-468 cells may be associated with biological enrichment pathways such as “JAK-STAT signaling pathway,” “NF-κB signaling pathway,” and “TNF signaling pathway” (Fig. 2E–N).

To further explore the consistency of the potential mechanism of action of MDA-MB-468 cells in various hyperlipidemic microenvironments, we established a new hyperlipidemic model, that is, a co-culture model of mouse 3T3-L1 and MDA-MB-468 cells. Concurrently, transcriptome sequencing revealed 858 upregulated and 768 downregulated genes (Fig. S1A, B). The KEGG pathway analysis indicated that co-cultured MDA-MB-468 cells might be involved in “Focal adhesion,” “ECM-receptor interaction,” “Cytokine-cytokine receptor interaction,” and “TNF signaling pathway,” with potential biological pathways and mechanisms, such as “Cytokine-cytokine receptor interaction” highlighted (Fig. S1C, D). Similarly, the GSEA indicated that the upregulated genes in MDA-MB-468 cells may be associated with biological enrichment pathways such as “Chemokine signaling pathways,” “NF-κB signaling pathways,” and “TNF signaling pathways” (Fig. S1E–N).

**Fig. 3:**
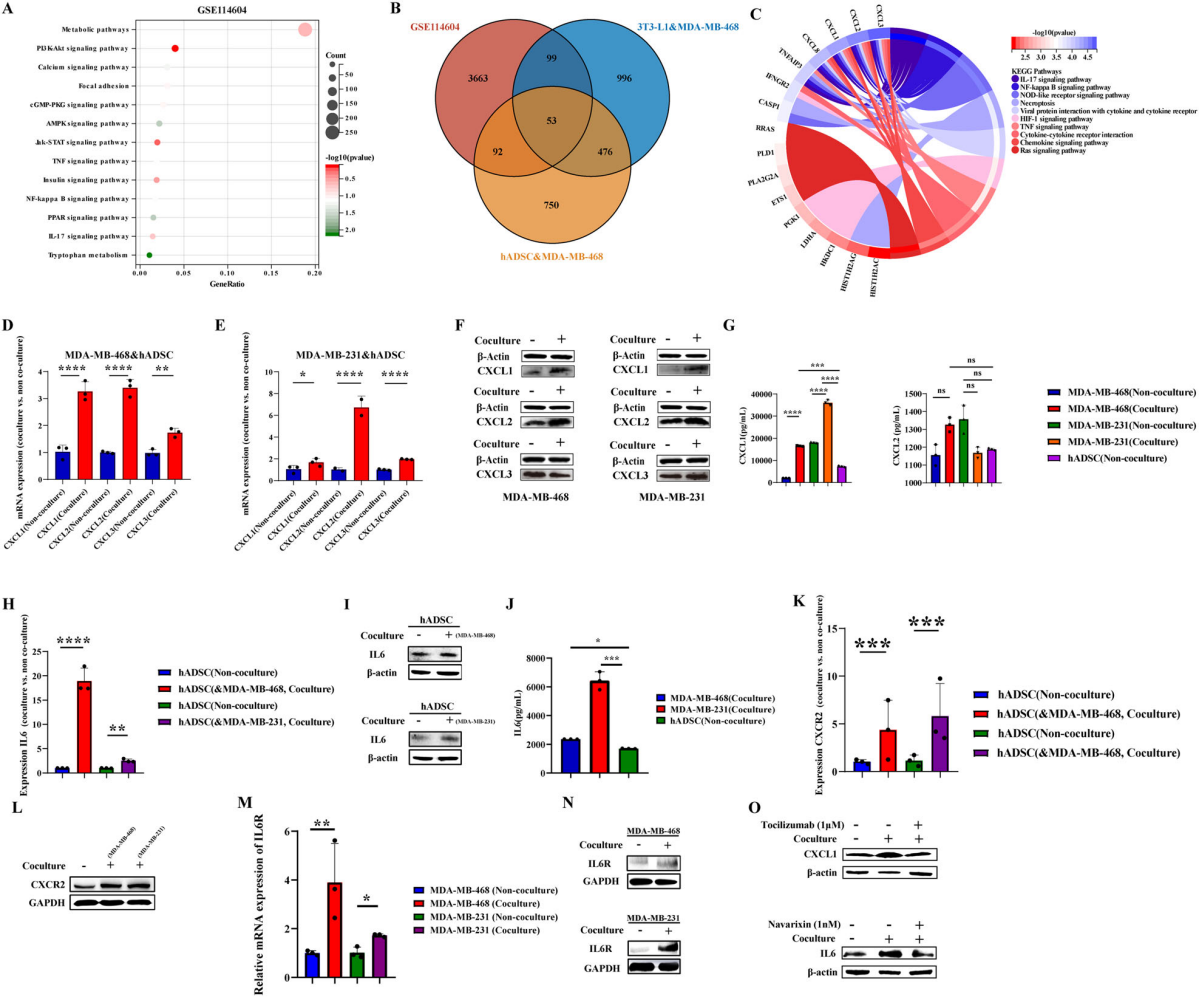
The analysis of the potential mechanism and the detection of the expression level of related genes and proteins in cocultured TNBC cells. MDA-MB-468 and MDA-MB-231 of TNBC cells were co-cultured with hADSC cells or cultured separately. After 3 days, the cells were collected for cell sequencing, RT-qPCR, ELISA and Western blotting. **A** The bubble diagram of the potential mechanism of co-cultured MDA-MB-231 cells was analyzed in GSE114604 data set; **B** Vanne diagram of differential gene intersection in GSE114604, hADSC&MDA-MB-468, and 3T3-L1&MDA-MB-468 datasets; **C** 53 differential overlapping gene enrichment analysis circles; **D**–**F** Detection of the gene and protein expressions of CXCL1, CXCL2 and CXCL3 in TNBC cells co-cultured with hADSC cells; **G** Detection of the secretion of CXCL1 and CXCL2 protein in cell culture medium after co-culture with hADSC cells; **H**–**J** Detection of the expression of IL6 gene and protein and the secretion of IL6 protein in hADSC cells co-cultured with TNBC cells. **K**, **L** Detection of the expression of CXCR2 gene and protein in hADSC cells co-cultured with TNBC cells. **M**, **N** Detection of the expression of IL6R gene and protein in TNBC cells co-cultured with hADSC cells; **O** MDA-MB-468 and MDA-MB-231 cells were cultured alone or co-cultured with hADSC cells. Add inhibitors for IL6R (Tocilizumab, 1 μM) and/or CXCR2 inhibitors (Navarixin, 1 nM), or PBS to the culture medium. 3 days later, detection of the expression of CXCL1 in TNBC cells and IL6 in hADSC cells.

Furthermore, we downloaded the GSE114604 dataset from the Gene Expression Omnibus database, which was the sequence of a tumor-bearing mouse model constructed using MDA-MB-468 cells in obese and non-obese mouse. The differential gene enrichment pathway analysis revealed that diet-induced obese mouse might be involved in the “PI3K-Akt signaling pathway,” “JAK-STAT signaling pathway,” and “NF-κB signaling pathway” (Fig. 3A).

Additionally, we comprehensively analyzed the potential mechanism of TNBC in a high-fat microenvironment by analyzing the intersection of three data sets: GSE114604, hADSC & MDA-MB-468, and 3T3-L1 & MDA-MB-468 datasets. The Venn diagram illustrated 53 differentially expressed genes intersecting in the sequencing results of the three databases (Fig. 3B). KEGG pathway analysis of these 53 genes suggested that potential mechanisms of TNBC in a high-fat microenvironment might involve the “IL-17 signaling pathway,” “NF-κB signaling pathway,” “TNF signaling pathway,” and “Chemokine signaling pathway” (Fig. 3C). Based on the database analysis, we hypothesized that the potential mechanism of TNBC in the hyperlipidemic microenvironment may be related to inflammation or the chemokine pathway.

### Up-regulation and secretion of CXCL1 in co-cultured TNBC cells and IL6 in co-cultured adipocytes

Co-culture with hADSC could promote the expression of *CXCL1*, *CXCL2*, and *CXCL3* genes in MDA-MB-468 and MDA-MB-231 cells; however, only the genes and protein expression of CXCL1 and CXCL2 was consistently upregulated in co-cultured MDA-MB-468 and MDA-MB-231 cells (Fig. 3D–F). On further investigation of the secretion levels of CXCL1 and CXCL2 in the co-culture supernatant, ELISA detection showed that the secretion of CXCL1 in the co-culture supernatant was upregulated compared to that in the non-co-culture system (Fig. 3G).

Adipocytes secrete adipokines, including IL6. In our study, we verified this by constructing two co-culture systems: hADSC & MDA-MB-468 and hADSC & MDA-MB-231. We confirmed this at the gene and protein levels, finding that IL6 expression in co-cultured adipocytes was higher than that in adipocytes alone (Fig. 3H, I). We further analyzed the level of IL6 secretion after co-culture and found that the level of IL6 secretion in the culture supernatant after co-culture was higher than that in the supernatant of non-co-cultured adipocytes (Fig. 3J).

### Upregulation of CXCR2 expression in co-cultured TNBC cells, IL6R expression in co-cultured hADSC

As CXCR2 is the binding receptor of CXCL1, we observed higher levels of CXCR2 expression in hADSC co-cultured with MDA-MB-468 and MBA-MB-231 cells than in hADSC alone. IL6R, the binding receptor of IL6, exhibited higher expression in MBA-MB-468 and MBA-MB-231 cells after co-culture compared to non-co-cultured MDA-MB-468 and MBA-MB-231 cells (Fig. 3K–N). In this study, Tocilizumab (1 μM), an IL6R neutralizing antibody, was used to reduce the expression of CXCL1 protein in MDA-MB-468 and MDA-MB-231 cells co-cultured with adipocytes. ed Navarixin (1 nM), a CXCR2 neutralizing antibody, was added to the co-culture system, and the inhibitor successfully reduced the expression of IL6 in hADSC co-cultured with TNBC cells (Fig. 3O).

### Activation of STAT3/NF- κB p65 signal pathway in co-cultured TNBC cells

**Fig. 4:**
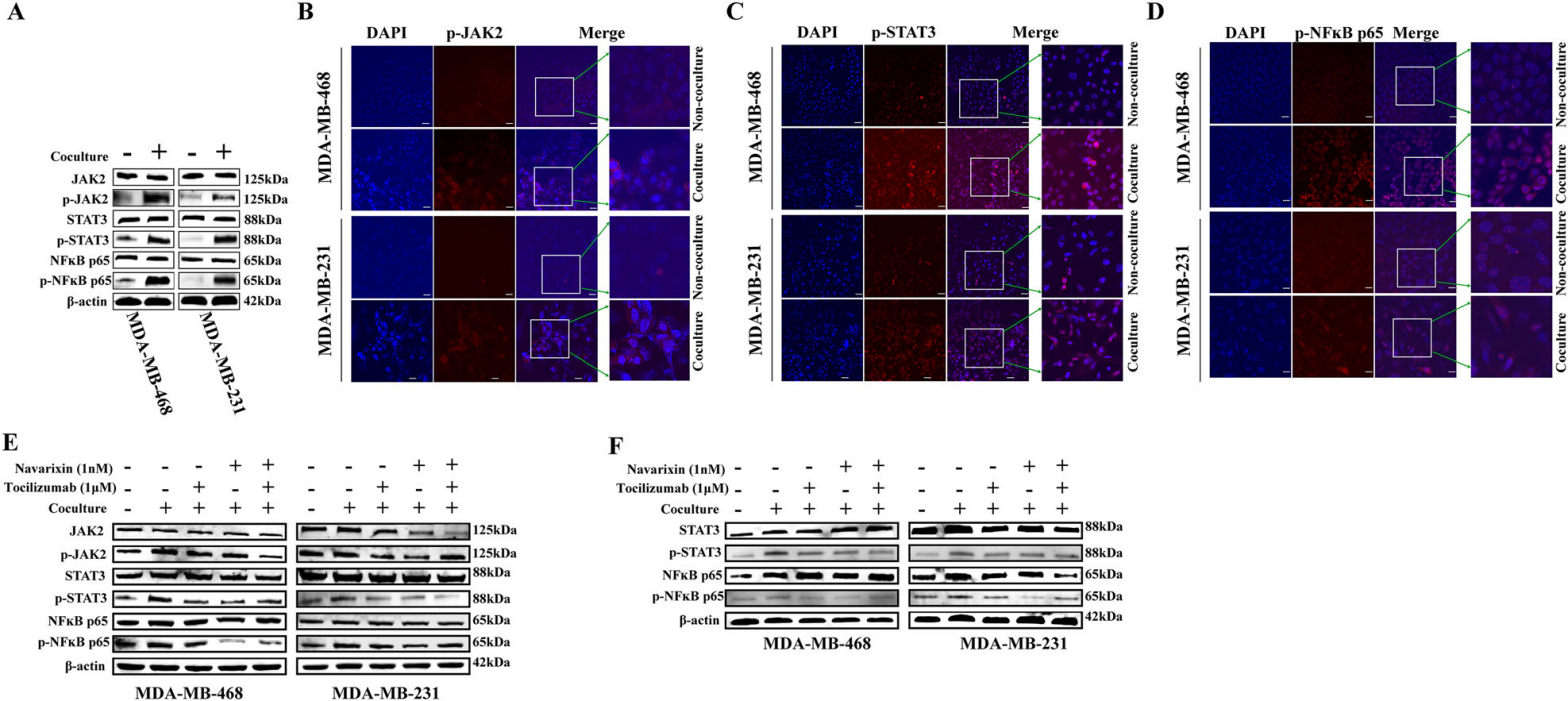
hADSC and TNBC cells co-cultured to activate STAT3/NF-κB p65 signal pathway in TNBC cells and hADSC cells. MDA-MB-468 and MDA-MB-231 of TNBC cells were co-cultured with hADSC cells or cultured separately. After 3 days, the cells were collected for Western blotting and cell immunofluorescence to detect the expression of related pathway proteins. **A** Western blotting was used to detect the expression of proteins related to STAT3/NF-κB p65 pathway in co-cultured TNBC cells; **B**–**D** The expression of proteins related to STAT3/NF-κB p65 pathway in co-cultured TNBC cells was detected by cellular immunofluorescence, MDA-MB-468 and MDA-MB-231 cells grew on the cover slides of the insert, respectively. The cells in the lower chamber were cultured alone or co-cultured with adipocytes. After 3 days, the cells were fixed and stained, and the expressions of P-JAK2, P-STAT3 and P-NF-κB p65 were detected. The nucleus was stained with DAPI. Scale bars, 20 μm; **E** MDA-MB-468 and MDA-MB-231 cells were cultured alone or co-cultured with hADSC cells. Add inhibitors for IL6R (Tocilizumab, 1 μM) and/or CXCR2 inhibitors (Navarixin, 1 nM), or PBS to the culture medium. 3 days later, TNBC cells were collected to detect the expression of proteins related to STAT3/NF-κB p65 pathway; **F** MDA-MB-468 and MDA-MB-231 cells were cultured alone or co-cultured with hADSC cells. Add inhibitors for IL6R (Tocilizumab, 1 μM) and/or CXCR2 inhibitors (Navarixin, 1 nM), and PBS to the culture medium. 3 days later, hADSC cells were collected to detect the expression of proteins related to STAT3/NF-κB p65 pathway.

Previous reports have indicated that adipokines from adipocytes can activate cell surface receptors and initiate signals through Janus kinase (JAK) or signal transducer and transcriptional activator (STAT) signal pathways. Combined with our sequencing results, we hypothesized that co-culture of hADSC and TNBC cells could activate the STAT3/NF-κB p65 signaling pathway in TNBC cells. Our observations that co-culture with adipocytes led to the activation of phosphorylation of JAK2, STAT3, and NF-κB p65 proteins in MDA-MB-468 and MDA-MB-231 TNBC cells (Fig. 4A). Additionally, we observed stronger fluorescence signals of phosphorylated JAK2, STAT3, and NF-κB p65 proteins in co-cultured TNBC cells (Fig. 4B–E).

### Transcriptional sequence analysis of hADSC cells

Further exploration of the potential mechanism of co-cultured hADSC revealed 391 upregulated and 122 downregulated genes (Fig. S2A, B). KEGG pathway enrichment of differential genes suggested the involvement of co-cultured hADSC cells in the biological enrichment processes and mechanism of the “HIF-1 signaling pathway,” “PI3K-Akt signaling pathway,” and “Breast cancer” (Fig. S2C). GSEA enrichment indicated that upregulated genes in co-cultured hADSC may be associated with biological enrichment pathways such as “Jak-STAT signaling pathway,” “NF-κB signaling pathway,” “PI3K-AKT signaling pathway,” and “HIF-1 signaling pathway” (Fig. S2D–M). Based on the sequencing results, we hypothesized that co-cultured adipocytes might play a role through the STAT3/NF-κB p65 signaling pathway. We observed an increase in the phosphorylation level of STAT3 and NF-κB p65 proteins after co-culture. The addition of Tocilizumab (1 μM), Navarixin (1 nM), and Tocilizumab (1 μM) and Navarixin (1 nM) could inhibit the STAT3/NF-κB p65 signaling pathway of Fig. 4F. Therefore, we hypothesized that there might be a mutually reinforcing process between TNBC cells and adipocytes in the high-fat microenvironment.

### WP1066 reduces the migration and invasion abilities of co-cultured TNBC cells and inhibits the STAT3/NF-κB p65 signal pathway

**Fig. 5:**
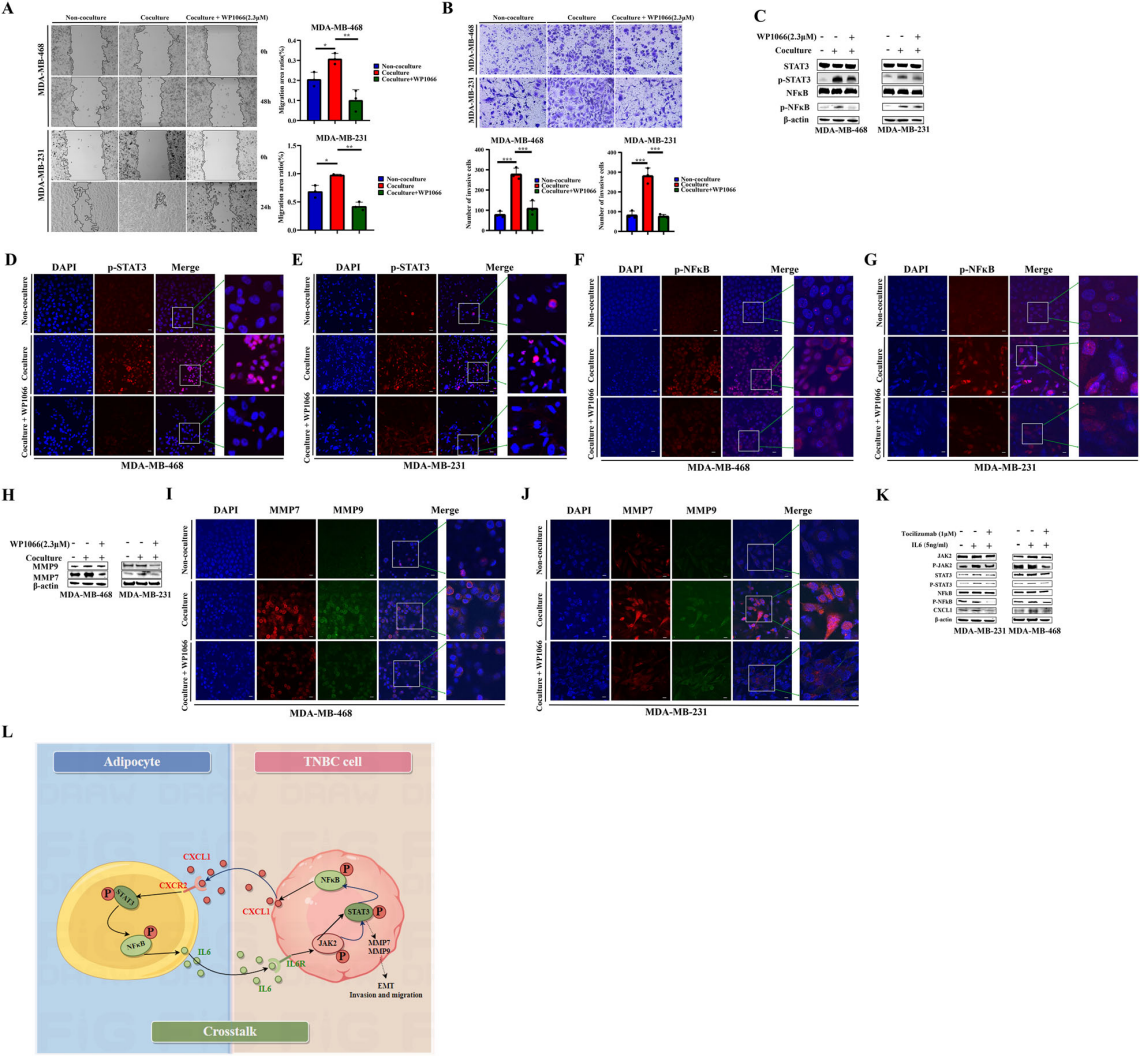
Co-culture with hADSC cells increased the expression of MMP7/MMP9 in TNBC cells and inhibited the STAT3/NF-κB pathway to weaken the invasion and migration ability of TNBC cells. MDA-MB-468 and MDA-MB-231 cells were cultured alone or co-cultured with hADSC cells. Add inhibitors for JAK (WWP1066, 2.3 μM) or PBS to the culture medium. **A**, **B** 3 days later, TNBC cells were collected to detect the migration and invasion abilities; **C**–**G** 3 days later, TNBC cells were collected to detect the expression of proteins related to STAT3 / NF-κB p65 pathway by Western blotting and immunofluorescence; **H**–**J** 3 days later, TNBC cells were collected to detect the expression of proteins of MMP7/MMP9 by Western blotting and immunofluorescence. **K** IL-6-mediated adipocyte microenvironment can activate STAT3/NF-κB pathway and regulate the expression of CXCL1. **L** Adipocyte-derived IL-6 and TNBC cell-derived CXCL1 co-mediate the interaction mechanism. Adipocyte-derived IL6 activates STAT3/NF-κB pathway in TNBC cells to promote the expression and secretion of CXCL1 in TNBC and promote tumor progression. Tumor-derived CXCL1 further activates STAT3/NF-κB pathway in adipocytes to promote IL6 expression and secretion in adipocytes, and finally forms a cascade of interaction.

WP1066 is an inhibitor of JAK2 and an upstream antagonist of STAT3. We suspected that WP1066 might affect the downstream signaling pathway or the expression of related protein molecules. Therefore, we constructed the co-culture model of MDA-MB-468 and MDA-MB-231 TNBC cells with adipocytes, and the treatment with WP1066 (2.3 μM) reduced the migration and invasion abilities of TNBC cells after co-culture (Fig. 5A, B). We also found that WP1066 (2.3 μM) could inhibit the phosphorylation of STAT3 and NF-κB p65 proteins in co-cultured MDA-MB-468 and MDA-MB-231 TNBC cells (Fig. 5C). The fluorescence signals of STAT3 and NF-κB p65 phosphorylated proteins in TNBC cells decreased upon the addition of WP1066 (2.3 μM) (Fig. 5D–G).

The expression levels of metalloproteinase 7 (MMP7) and MMP9 in TNBC cells co-cultured with adipocytes were higher than those in non-co-cultured cells, potentially explaining the increased migration and invasion abilities of TNBC cells co-cultured with adipocytes. Treatment with WP1066 (2.3 μM) decreased the expression of MMP7 and MMP9 in co-cultured TNBC cells (Fig. 5H). The fluorescence signals of MMP7 and MMP9 expression proteins in TNBC cells decreased with the addition of WP1066 (2.3 μM) (Fig. 5I).

### Effects of Recombinant IL6 and Tocilizumab Inhibitors on STAT3- NF κB p65 Signal Pathway in TNBC cells

To investigate whether IL6 can activate STAT3- NF-κB p65 signal pathway in TNBC cells, recombinant IL6 was added to the cell culture medium. We found that recombinant IL6 (5 ng/mL) can promote TNBC cell migration and invasion (Fig. S3). Exposing TNBC cells to exogenous IL6 (5 ng/mL) activated the phosphorylation of JAK2, STAT3, and NF-κB p65 proteins in MDA-MB-468 and MDA-MB-231 TNBC cells. The addition of recombinant IL6 (5 ng/mL) led to an increase in CXCL1 protein expression in MDA-MB-468 and MDA-MB-231 TNBC cells. When treated with Tocilizumab (1 μM), this process was inhibited, resulting in a decrease in the phosphorylation of JAK2, STAT3, and NF-κB p65 proteins as well as a decrease in CXCL1 expression in MDA-MB-468 and MDA-MB-231 TNBC cells (Fig. 5J).

Above all, we speculate that there is a close relationship between adipocytes and TNBC cells, forming an interaction network. Adipocyte-derived IL6 activates STAT3/NF-κB pathway in TNBC cells to promote the expression and secretion of CXCL1 in TNBC and promote tumor progression. Tumor-derived CXCL1 further activates STAT3/NF-κB pathway in adipocytes to promote IL6 expression and secretion in adipocytes, and finally forms a cascade of interaction (Fig. 5L).

### Obesity induced by HFD promotes tumor growth in C57 BL/6J mouse

**Fig. 6:**
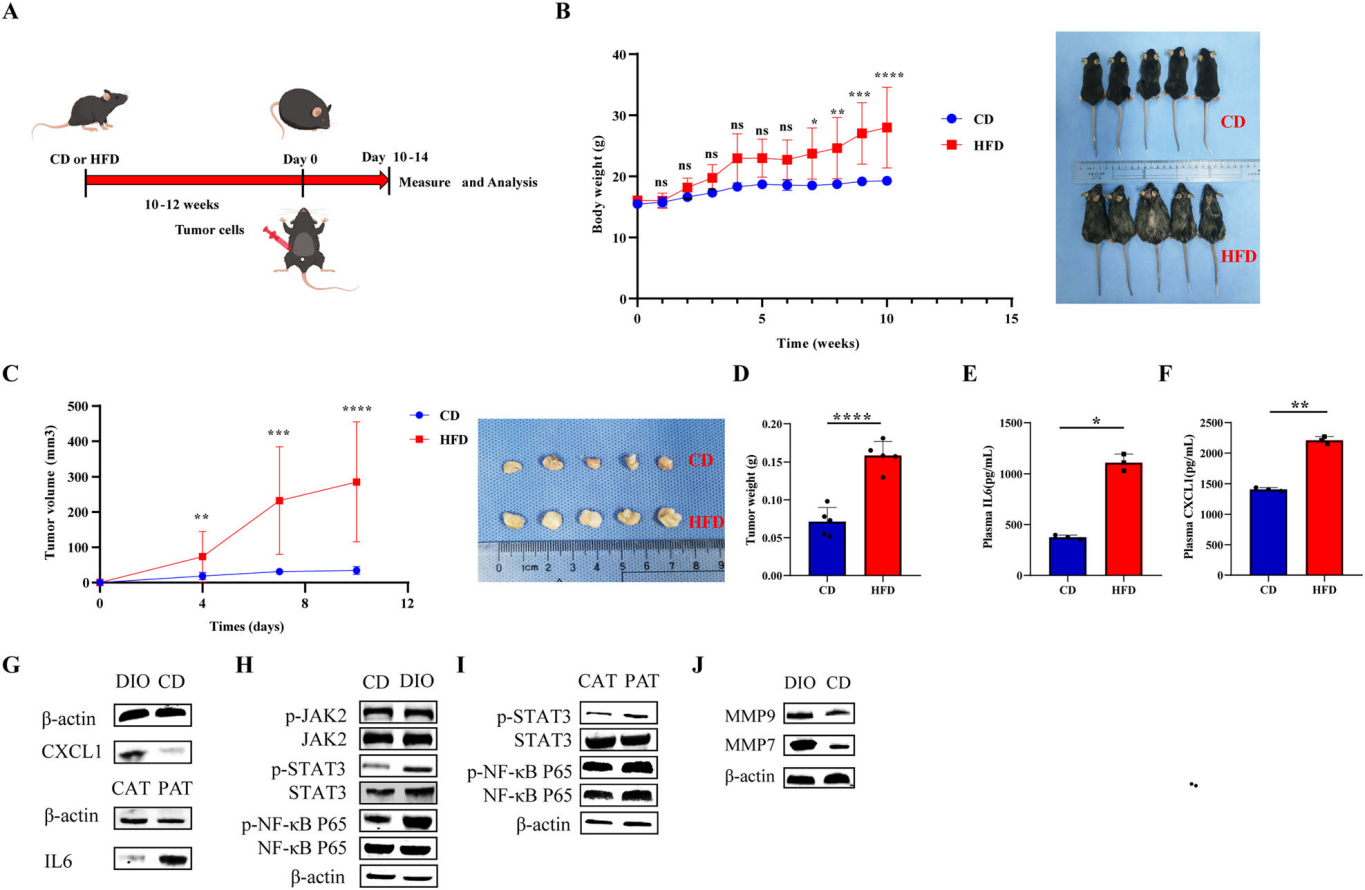
Diet-induced obese model mouse increased tumor-bearing load. **A** Female C57BL/6J mouse of HFD-induced obesity model were fed with high-fat diet or controlled diet for 10–12 weeks, and a total of 4 × 10^6^ MDA-MB-468 cells/200 μL was injected into each C57BL/6J mouse fat pad for 10–14 days; **B** The body weight of HFD mouse increased significantly; **C**, **D** The tumor growth rate and tumor load of HFD mouse was faster than that of CD mouse; **E**, **F** Detection of the secretion levels of serum CXCL1 and IL6 of mouse after tumor bearing; **G** Western blotting was used to detect the expression of CXCL1 in tumor tissue and IL6 in adipose tissue; **H**–**J** Western blotting was used to detect the expression of proteins related to STAT3/NF-κB p65 pathway in tumor tissue and adipose tissue; **J** Western blotting was used to detect the expression of MMP7/MMP9 in tumor tissue of DIO and CD mouse.

Mouse were fed an HFD for 10–12 weeks to induce obesity, followed by the implantation of TNBC cells in the mammary glands of mouse (Fig. 6A, B). Tumor load was higher in obese mouse, and tumors grew faster compared to control mouse (Fig. 6C, D). The levels of IL6 and CXCL1 in the serum of HFD mouse were significantly higher compared to the LFD group (Fig. 6E, F). Additionally, CXCL1 expression in tumor tissues of HFD mouse was higher than that in LFD mouse (Fig. 6G). We investigated the differential expression of IL6 between peri-tumor infiltrating adipose tissue and contralateral adipose tissue in HFD mouse. Western Blotting revealed higher IL6 expression in the infiltrated adipose tissue around the tumor in HFD mouse compared to the contralateral adipose tissue of the tumor (Fig. 6G). To investigate the potential mechanism of tumors in HFD mouse, we found that HFD mouse could activate the phosphorylation of JAK2, STAT3, and NF-κB p65 protein in TNBC tumor tissue (Fig. 6H). We also analyzed the mechanism of adipose infiltration around the tumor tissues of HFD mouse. In obese mouse, compared with the contralateral adipose tissue of the tumor, the ipsilateral adipose tissue of the tumor activated the STAT3/NF-κB p65 signal pathway and increased IL6 secretion (Fig. 6I). Western Blotting also demonstrated an increased expression of MMP7/MMP9 in TNBC tumor tissues of obese mouse in the HFD group (Fig. 6J). All uncropped Western blots were uploaded in Original Data.

## Discussion

Studies have shown that a special type of fat cells (CAAs) exists in the matrix surrounding invasive BC. Compared with normal adipocytes, this type of adipocytes has a series of characteristics such as a fibroblast-like phenotype, small size, small and scattered lipid droplets, over-expression of VI collagen, and low expression of adiponectin (APN) and other lipofactors [29]. CAA-BC cell cross-talk can promote the progression and metastasis of breast cancer by secreting a variety of fat factors and plays a role in tumor feature remodeling. Close proximity between BC cells and adipocytes induces changes, giving rise to CAA with unique phenotypic, gene expression, and secretory characteristics [25, 27]. Co-culture of tumor cells with adipocytes enhances invasive potential, and both cell types exhibit metabolic adaptations supporting tumor progression [30]. However, in literature, the impact of tumor cells on adipocytes has rarely been reported, and the interaction mechanism between them is still unclear.

The cross-talk between adipocytes and BC cells, mediated by adipokines secreted by adipocytes, promotes breast cancer proliferation, survival, and metastasis [31]. Under pathological conditions, such as obesity and cancer, the level of IL6 secreted by adipocytes significantly increases. Changes in adipocyte secretion profiles (such as IL6) were observed in co-cultured BC cells or isolated adipocytes [32]. Lee et al. [33] indicated that 3T3-L1 adipocytes co-cultured with BC cells could upregulate the expression of the inflammation-related IL6 and PTX3, consistent with the overexpression of IL6 in human BC tissue CAAs. Fujisaki et al. [34] isolated normal breast adipocytes and CAAs, co-cultured them in a collagen gel to simulate an in vivo environment, and found that IL6 was higher in the medium under CAA conditions. Similarly, adipocyte-derived IL6 secretion increases when adipocytes are co-cultured with MDA-MB-231 TNBC cells in vitro, whereas blocking IL6 significantly reduces the size and number of nodules in a TNBC lung metastasis model [35]. In our study, we found that adipocytes secrete IL6, and the secreted IL6 can bind to IL6R in MDA-MB-468 and MDA-MB-231 cells, thereby contributing to the poor prognosis of TNBC cells.

To further investigate the interaction between adipocytes and TNBC cells after co-culture, we selected MDA-MB-468 cells to co-culture with 3T3-L1 and hADCS cells separately, analyzing the outcomes through gene sequencing. The results revealed that the co-cultured TNBC cells might be associated with the “JAK-STAT signaling pathway” and “NF-κB signaling pathway.” Moreover, we obtained data for analysis from the GSE114604 dataset of the GEO database. GSE114604 data mainly analyzed the sequencing results of TNBC cells after the co-culture of MDA-MB-231 cells and adipocytes. The results also indicated that it was related to the JAK-STAT and “NF-κB signaling pathways.” This data led us to hypothesize that IL6 secreted by adipocytes could activate the JAK/STAT3 signal pathway when interacting with the IL6R receptor of TNBC cells. The IL6/JAK/STAT3 signaling pathway is a classical pathway that influences the onset and progression of breast cancer. Classical IL6 signaling involves strict binding to the membrane-binding receptor IL6R, increasing its affinity for transmembrane gp130 [36]. This effect is mediated by JAK/STAT3 phosphorylation [37, 38]. In female breast cancer, IL6 can mediate the interaction between the TME and tumor cells. In a high-fat microenvironment, IL6 produced by fat acts on tumor cells [39]. In pathophysiology, IL6 mediates inflammation and regulates the carcinogenic pathway of JAK/STAT3 [40]. The JAK and STAT proteins, especially STAT3, are promising targets for cancer therapy. STAT3 is a transcriptional activator and oncogene tightly regulated under physiological conditions. STAT3 is constitutively activated in all breast cancer subtypes but mainly in triple-negative cancer [41]. The activated JAK phosphorylates the receptor on the membrane. The STAT3 protein binds to the phosphorylated tyrosine residue of the receptor through the SH2 domain, and phosphorylates the Y705 residue under the action of JAK, thereby achieving activation and forming a dimer. After the dimerized STAT3 is separated from the receptor, it enters the nucleus, binds to the DNA response element of the target gene, regulating the transcription and expression of the target gene [42]. STAT3 is the main downstream regulator of IL6 signal transduction and plays a unique role in regulating inflammation and tumor transformation [43, 44]. IL6 promotes tumor cell proliferation and angiogenesis by regulating the JAK/STAT3 signaling pathway [18]. In HER2-positive BC, IL6 induces the production and maintenance of BC stem cells through the NF-κB p65 and STAT3 signal pathways, promoting tumor progression [45]. Detected STAT3 activity in BC cells is associated with tumor cell migration [46]. Additionally, in T47D cells, the IL6/STAT axis stimulates migration [47].

The use of the IL6R inhibitor tocilizumab resulted in the inhibition of the JAK/STAT3 pathway in TNBC cells. Consequently, the proliferation and migration abilities of TNBC cells decreased upon treatment with the downstream JAK/STAT3 inhibitor WP1066. We further validated this result by adding recombinant IL6 protein, which activated the JAK/STAT3 pathway. Blocking IL6R using an IL6R antibody reversed IL6-induced breast cancer metastasis [48, 49]. We selected downstream JAK/STAT3 inhibitors to validate their invasion and migration abilities to further demonstrate the role of this classical inflammatory pathway in the regulation of TNBC invasion and migration. These results indicated that the IL6/STAT3 was important in the interaction between adipocytes and TNBC cells. Similarly, the addition of IL6 recombinant protein can activate STAT3/NF-κB p65 signal pathway in TNBC cells, while IL6R inhibitor Tocilizumab can inhibit this process. NF-κB activation is a well-studied transcription factor controlling various cellular processes in cancer, including inflammation, transformation, invasion, proliferation, metastasis, angiogenesis, chemotherapy resistance, and radiation resistance [50]. Chung et al. revealed the protein-protein interaction between STAT3 and NF-κB in human breast cancer cells by immunoprecipitation [51]. Similarly, Yoshida et al. have also observed that NF-κB p65 can cooperate with non-tyrosine phosphorylated STAT3 and be activated by IL1 [52]. Activation of NF-κB and STAT3 can control the expression of anti-apoptosis, proliferation, and immune response genes. STAT3 plays an important carcinogenic role in malignant and precancerous cells and may also inhibit tumor promotion through its anti-inflammatory effects in inflammatory cells. Other interactions and cross-talk forms between NF-κB and STAT3 include physical interactions, with these factors cooperating on gene promoters or enhancers. Together, NF-κB and STAT3 cooperate to promote the occurrence and progression of colon, gastric, and hepatocellular carcinoma [53]. IL6, when binding to a soluble or membrane-bound IL6R polypeptide, signals through the interaction with membrane-related gp130 subunits. This triggers the activation of JAKs and downstream effectors such as STAT3, Shp2-Ras, and phosphatidylinositol 3-kinase (PI3K)-Akt, which in turn influence the expression of NF-κB [54, 55]. Therefore, we speculate that when we inhibit the JAK/STAT3 pathway, the expression of p-NF-κB p65 protein can also be inhibited, and the expression of p-NF-κB p65 may be regulated by the JAK/STAT3 pathway.

In this study, we found that the expression and secretion of CXCL1 increased in co-cultured TNBC cells. Previous studies have reported that the expression of CXCL1 is related to the poor prognosis of BC (including TNBC) [56–58]. Yang et al. [59] found that chemokine CXCL1 stimulates the migration and invasion of ER-negative BC cells by activating the ERK/MMP2/9 signaling axis. Previous studies indicated that CXCL1 is upregulated in the plasma and stroma of BC patients [60, 61]. CXCR2 is the receptor of CXCL1. We found that the level of CXCR2 in the co-cultured adipocytes increased after sequencing and verification by PCR and WB. Zhang et al. studied the interaction between adipose stromal cells and cancer cells in the TME. They showed that adipose stromal cells can migrate to chemokines CXCL1 and CXCL8 through the chemokine receptors CXCR1 and CXCR2. The binding of CXCL1 and CXCR2 can be influenced by the NF-κB pathway, impacting the proliferation and spread of tumor cells [62]. Xu et al. found that NF-κB-mediated CXCL1 production plays a role in the maintenance of bone cancer [63]. Hartman et al. [64] observed that in TNBC cells, NF-κB signal cascade can affect the expression of chemokine CXCL1, and CXCL1 can bind to the transcription factor binding site of NF-κB. Inhibition of NF-κB results in the blockade of CXCL1 expression and production. Therefore, we speculated that CXCL1 derived from TNBC cells can act on adipocytes and that CXCL1 expression is regulated by NF-κB p65. The use of IL6R inhibitors resulted in the inhibition of CXCL1 expression in TNBC cells.

Conducting gene sequencing and pathway enrichment analyses on differentially expressed genes in adipocytes, we found that the co-cultured adipocytes may be regulated by the “JAK-STAT signaling pathway” and “NF-κB signaling pathway.” We also found that co-cultured adipocytes could activate the upregulation of p-STAT3 and p-NF-κB p65, thus impacting the synthesis and production of IL6. NF-κB-induced IL6 is implicated in tumor development and growth, primarily by increasing the survival and growth of cancer cells, as well as influencing the immune system to sustain tumor-associated inflammation [55, 65]. CXCR2 inhibitors inhibit the expression of p-STAT3 and p-NF-κB p65 in adipocytes. Our findings indicate that CXCL1 binding to CXCR2 activates the STAT3/NF-κB p65 pathway, subsequently affecting IL6 expression and secretion.

CAAs have been reported to produce various proteases that promote cancer cell invasion and metastasis. In breast cancer, CAAs express high levels of MMP11, promoting cancer cell invasion into surrounding tissues [66, 67]. Short-term co-culture of cancer cells and adipocytes induces upregulation of MMP2 in MCF7 cells, enhancing their invasiveness [68]. MMP2 and MMP9 produced by tumor cells are controlled by adipocyte-derived leptin and IL6 by activating FAK- and SRC-dependent pathways [69]. Given the enhanced invasion and migration ability of TNBC cells after co-culture, we hypothesized that TNBC cells might be affected by the change in EMT after co-culture with adipocytes. Therefore, we detected the expression levels of EMT-related gene expression proteins MMP7 and MMP9. We observed that the expression levels of MMP7 and MMP9 in TNBC cells increased after co-culture. The activation of STAT3 may regulate the expression of EMT-related genes in co-cultured TNBC cells. This finding aligns with previously published data on the effect of adipocytes on the proliferation, migration, and invasion of BC cells and confirms the role of IL6 in tumor invasion and migration [68, 70, 71]. Blocking the IL6 signal in BC cells and adipocytes induces alterations in the expression of EMT regulatory genes, disrupting local adhesion and reducing cell viability, thus reducing the proliferation, migration, and invasion of BC cells [72]. Nevertheless, we found that the expression of MMP7 and MMP9 increased after co-culture, and the use of IL6R or JAK/STAT3 inhibitors reduced the expression of MMP7/MMP9, consequently inhibiting the invasion and migration of TNBC cells. Similarly, we found that the tumor load in HFD-induced obese mouse was significantly higher than that in LFD mouse. The results observed in mouse were consistent with the mechanism verification of our in vitro experiments.

Our research has several limitations that should be noted. First, we used a hADSC line rather than primary adipocytes isolated from human adipose tissue. Future studies should incorporate primary cell cultures to improve the reliability of the findings. Second, our IL6 dose-response validation was limited to a single concentration. Including multiple concentration groups would strengthen the credibility of the results.

In conclusion, this study found that there is a cross-talk between TNBC cells and adipocytes, which can activate the STAT3 and NF-κB p65 pathways by producing and secreting CXCL1 and IL6, respectively. This signaling cascade ultimately contributes to the progression of TNBC. Blocking the signal communication between TNBC cells and adipocytes may provide a potential therapeutic strategy for TNBC.

## Materials and methods

### Cell lines and culture conditions

TNBC cells (MDA-MB-468 and MDA-MB-231) and mouse 3T3-L1 preadipocytes were purchased from the Cell Resource Center of the Shanghai Institute of Biochemistry and Cell Biology, Chinese Academy of Sciences. The cells were cultured in DMEM containing 10% fetal bovine serum (FBS), streptomycin (50 μ/mL), and penicillin (50 μ/mL). hADSC was purchased from Saibai Kang (Shanghai) Biotechnology Co., Ltd. hADSC was cultured in DMEM/F12 medium containing 10% FBS, streptomycin (50 μ/mL), and penicillin (50 μ/mL). All cells were cultured and maintained in a humidified 5% CO_2_ atmosphere at 37 °C.

The fused 3T3-L1 or hADSC cells were induced to differentiate into lipids in a differentiation medium (DM) containing 10% FBS, 0.5 mM 3-isobutyl-1-methylxanthine, 1 μM dexamethasone, and 10 μg/mL insulin for 6 days. Subsequently, the medium was replaced with DMEM adipocyte maintenance medium containing 10% FBS and 10 μg/mL insulin for 6 days, and the liquid was changed every 3 days (Fig. 1A).

Oil Red O staining was performed followed the manufacturer’s instructions. The differentiated adipocytes were washed with PBS (2–3 times), fixed with 4% paraformaldehyde for 30 min, washed with PBS (2–3 times), and stained with freshly prepared Oil Red O working solution at room temperature for 20 min. Subsequently, the stained adipocytes were cleaned and photographed under a microscope (Fig. 1B).

### Coculture, migration, and invasion assay

Twelve days after induction, adipocytes were co-cultured with TNBC cells (MDA-MB-468 and MDA-MB-231) using the Transwell culture system (0.4 μm pore diameter, Corning). The MDA-MB-468 and MDA-MB-231 cells were inoculated into the upper cavity. TNBC cells and adipocytes were co-cultured for 3 days, while MDA-MB-468 and MDA-MB-231 cells cultured alone were classified into the control group. The treatment and follow-up experiments were performed simultaneously (Fig. 1A).

To evaluate changes in the invasion and migration abilities of co-cultured TNBC cells, we isolated co-cultured TNBC cells for scratch and invasion experiments. We planted the two groups of cells in a twelve-well plate, placed in a complete medium containing 10% FBS for culture, waited for the cells to have iron walls, and removed and separated, and kept photos at different times. Both co-cultured and control tumor cells were seeded to observe cell healing and calculate the healing area of the cells. The upper chamber of the transwell chamber was covered with a Matrigel matrix (Corning, USA), and the tumor cell suspension was placed in the supraventricular hole and migrated to 20% FBS in the lower compartment. Subsequently, the migrating cells were fixed, stained, photographed, and counted using ImageJ software.

### Transcriptome sequencing

Total RNA was extracted from co-cultured MDA-MB-468 and non-co-cultured MDA-MB-468 cells, and cDNA and PCR were synthesized and sequenced. Differences in the expression levels of genes and transcripts between the two groups were calculated and analyzed, and enrichment analysis of the differentially expressed genes was performed.

### RNA extraction and quantitative real-time PCR

Total RNA was extracted using TRIzol ®Reagent (Invitrogen), and cDNA synthesis was performed with the PrimeScriptTM RT reagent Kit with gDNA Eraser (Perfect Real Time) (Takara) kit. All PCR analyses were conducted using qRT-PCR with FastStart Universal SYBR Green Master (ROX) (Roche) on an ABI QuantStudio fluorescence quantitative PCR. The relative mRNA expression of each gene was standardized with the internal control (β-actin or GAPDH). Primer sequences utilized in this study are shown in Table S1. qPCR analysis results are analyzed by the 2^–ΔΔ CT^ method.

### Western blotting

The cells were lysed to extract the total protein. The total protein and protein buffer were mixed in proportion and denatured in a 100 °C water bath for 10 min. The proteins were then separated using Tris-MOPS-SDS and transferred onto a PVDF membrane. The first antibody was incubated overnight at 4 °C, and the fluorescent antibody was incubated at room temperature for 1 h. Then, it was inserted into an image scanner. The image was scanned and saved. The antibodies used in this study are shown in Table S2.

### Immunofluorescence staining

TNBC cells were inoculated onto slides and cultured with or without adipocytes for 3 days. The cells were fixed in 4% paraformaldehyde for 20 min at room temperature and permeabilized in a 0.3% Triton Xmuri 100 solution for 15 min at room temperature. The cells were then sealed with 2% bovine serum albumin in PBS for 30 min. The first antibody was incubated overnight at 4 °C. The cells were then incubated with the appropriate secondary antibodies at room temperature for 1 h. The nuclei were stained with DAPI (Zhongshan Jinqiao, Beijing, China) and captured using a confocal microscope.

### Measurement of secreted adipokines and cytokines

After culturing with adipocytes alone or co-culturing for 3 days, the cell culture medium was centrifuged at 4 °C at 5000–10,000 rpm for 10 min, filtered with a 0.4 μm syringe filter, and stored at –80 °C. The secretion levels of CXCL1, CXCL2, CXCL3, and IL6 were detected. The ELISA was performed according to the manufacturer’s instructions. Each experiment was performed using the standard curve (SC).

### Recombinant human IL6 protein

Human recombinant IL6 protein (5 ng/mL; Proteintech, China) was used to treat TNBC cells for 48 h.

### Orthotopic xenograft tumor model

The animal experiments were conducted with approval from the Ethics Committee of Beijing Shijitan Hospital, affiliated with Capital Medical University [sjtky-lx-2022(045)], and adhered to the committee’s regulations. Four-week-old female C57BL/6J mouse were randomly classified into two groups and fed in isolation facilities to adapt to the environment for 1 week. The obese mouse group was fed a 60% fat high-fat diet (HFD) (D12492, Research Diets, USA), while the control group was fed a 10% fat low-fat diet (LFD) (D12450J, Research Diets, USA). After the obese mouse model was successfully established, MDA-MB-468 cells (4 × 10^6^/mouse) were injected in situ into the groin fat pad of the fourth mammary gland of both groups. The suspension was prepared according to the ratio of serum-free DMEM to VitroGel hydrogel solution (1:2). Tumor volume was measured using a caliper, calculated using the formula: *V* = (*a* × *b*^2^)/2 (*V* = tumor volume, *a* = maximum tumor diameter, and *b* = minimum tumor diameter). Blood samples from tumor-bearing mouse, tumor samples from mouse, and ipsilateral and contralateral adipose tissues from obese mouse were collected.

### Statistical analysis

The body weights and tumor volumes of the mouse were analyzed using two-way ANOVA. Continuous data were analyzed using the Mann–Whitney test (mean ± standard deviation), and differences between groups were analyzed using the unpaired Student’s *t*-test. All data were independently tested at least thrice. In the analysis of sequencing results, we selected “Fold Change” ≥2 and *p*-value < 0.05 as our criteria for screening differentially expressed genes. All statistical analyses were performed using the IBM SPSS Statistics software (version 25.0; Chicago, IL, USA). All double-tailed *p* < 0.05 were considered statistically significant. Among them, * indicated statistical significance at *p* < 0.05, ** indicated statistical significance at *p* < 0.01, *** indicated statistical significance at *p* < 0.001, and *** indicated statistical significance at *p* < 0.0001.

## Supplementary information

Original Data

Supplementary Figure legends

Figure S1

Figure S2

Figure S3

Table S1

Table S2

## Data Availability

The datasets used and/or analyzed in the current study are available from the corresponding author upon reasonable request.
