# Supplementary material for: Adipocyte-derived IL6 and triple-negative breast cancer cell-derived CXCL1 co-activate STAT3/NF-κB pathway to mediate the crosstalk between adipocytes and triple-negative breast cancer cells
Source: Cell Death Discov. 2025 Aug 21;11:395. doi: 10.1038/s41420-025-02713-4 (PMC12370983; doi:10.1038/s41420-025-02713-4)

Figure 3F

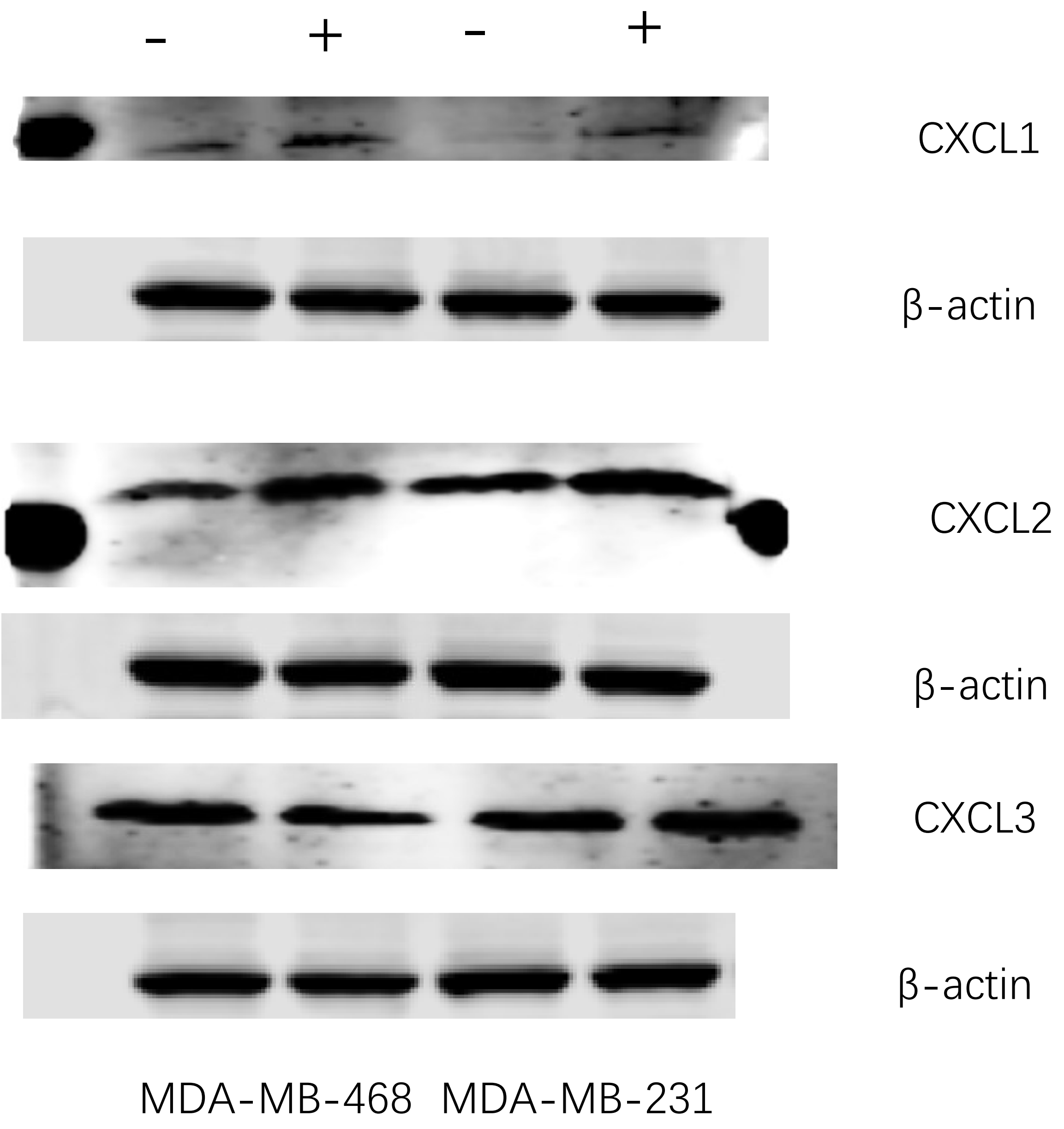

Figure 3L

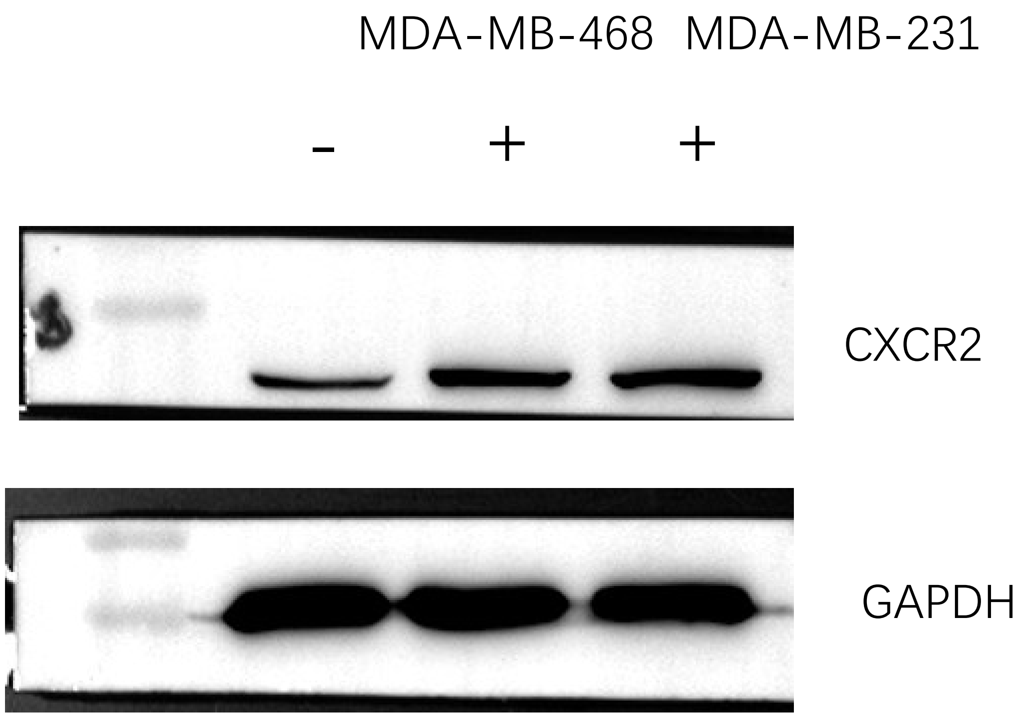

Figure 3N

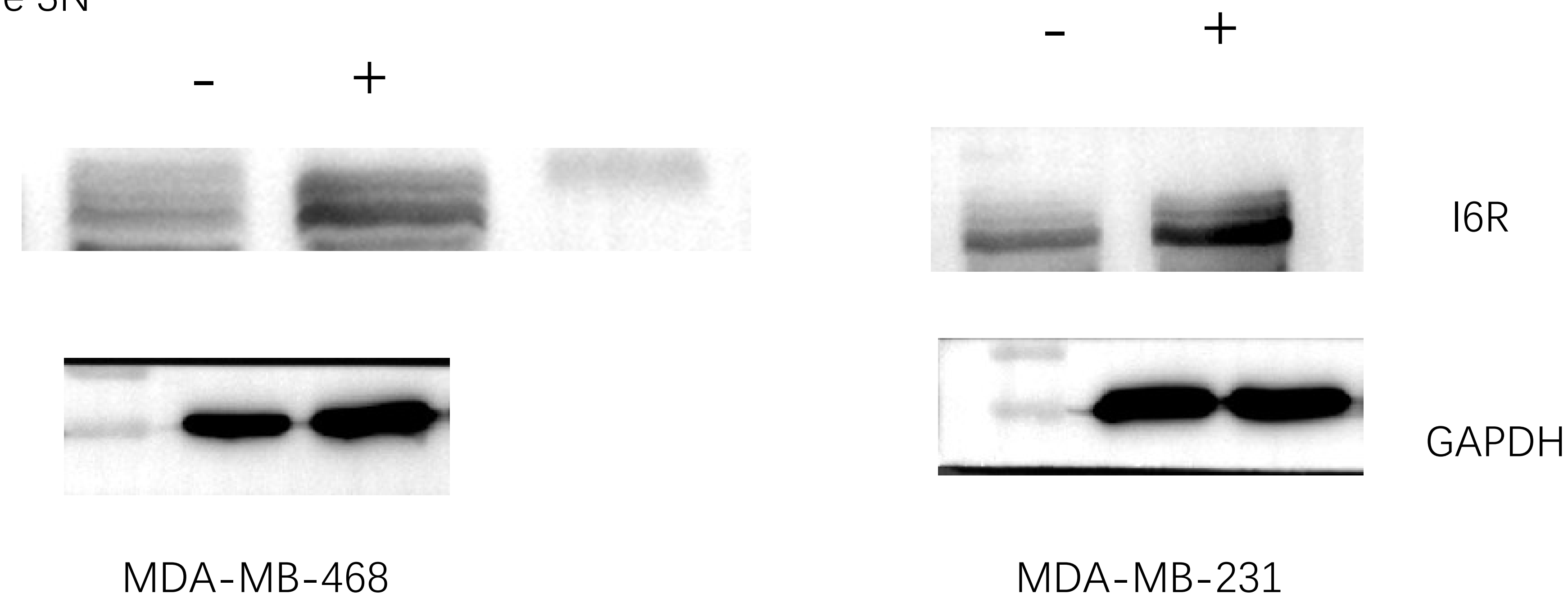

Figure 3O

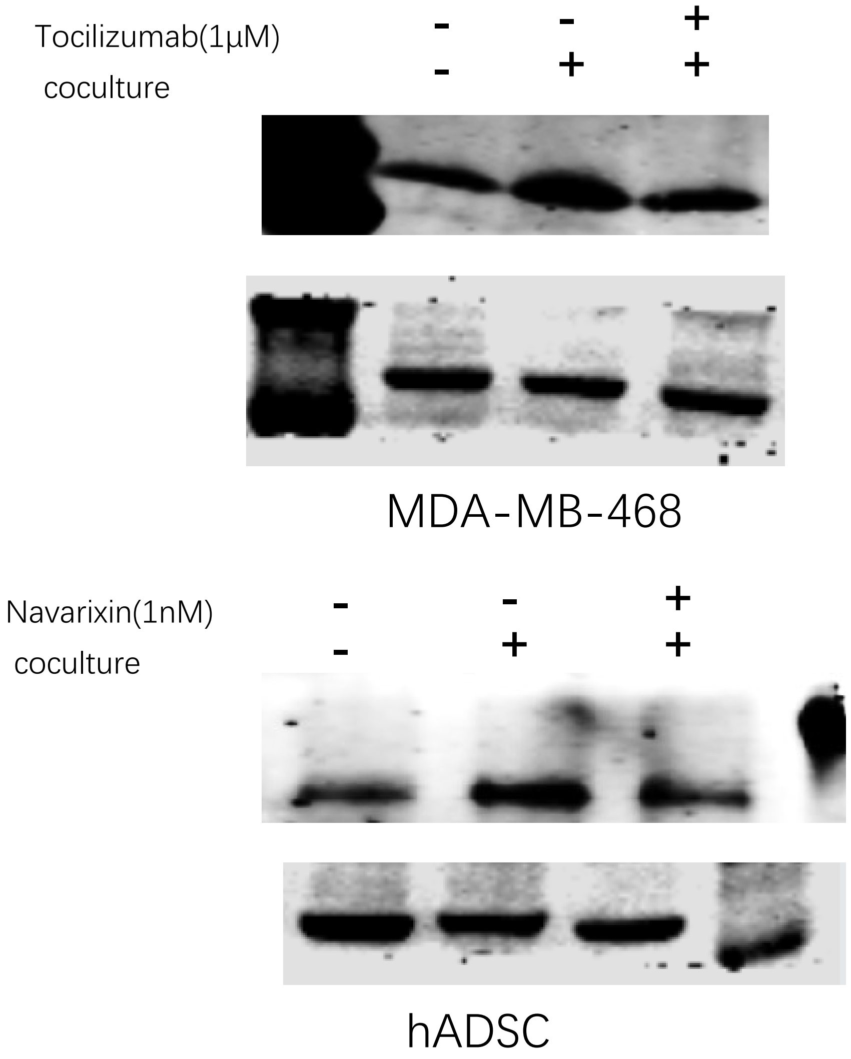
Tocilizumab(1μM)
coculture
- - +
- + +
MDA-MB-468
Navarixin(1nM)
coculture
- - +
- + +
hADSC

Figure 4A

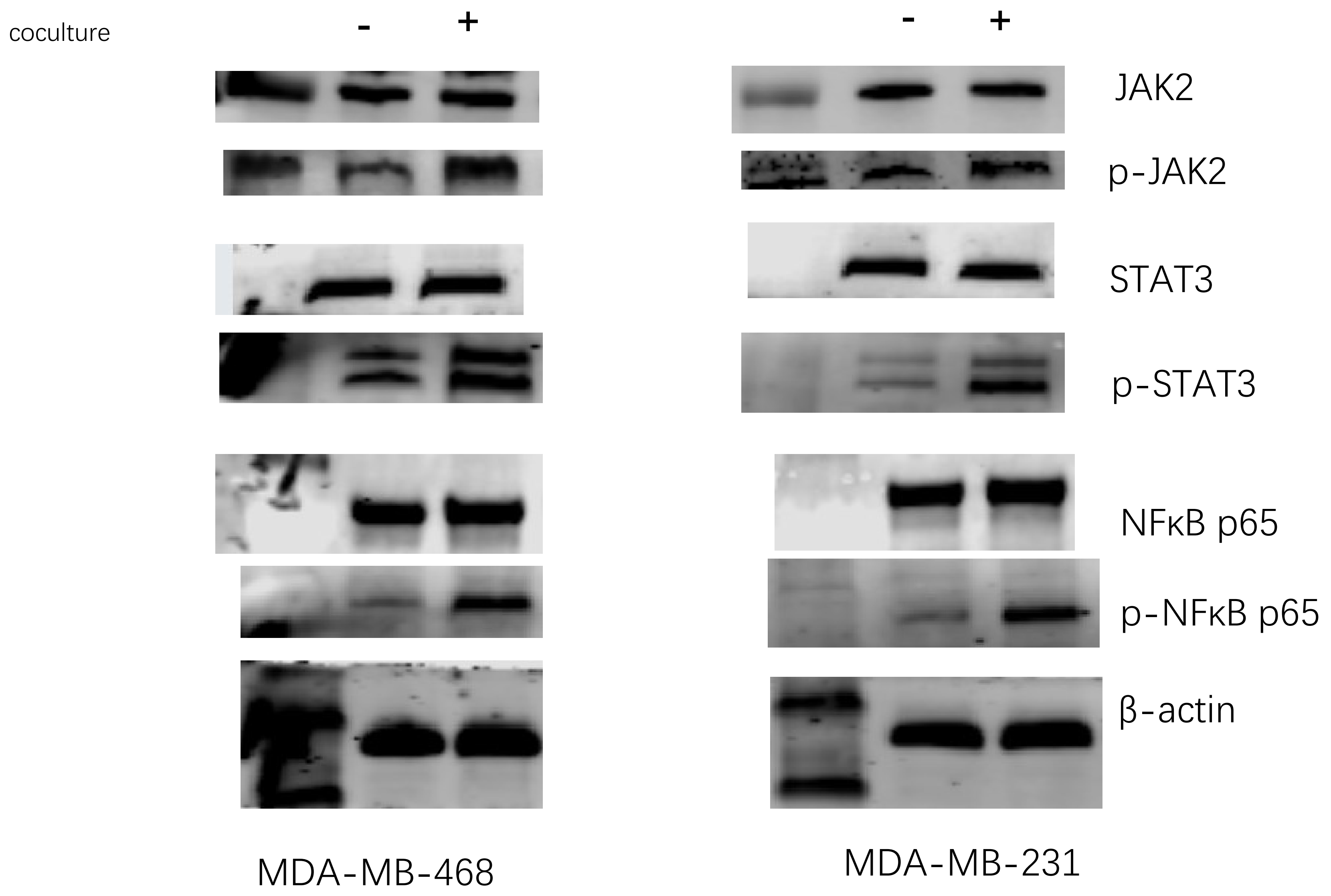

coculture
-
+
-
+
JAK2
p-JAK2
STAT3
p-STAT3
NFκB p65
p-NFκB p65
β-actin
MDA-MB-468
MDA-MB-231

Figure 4E

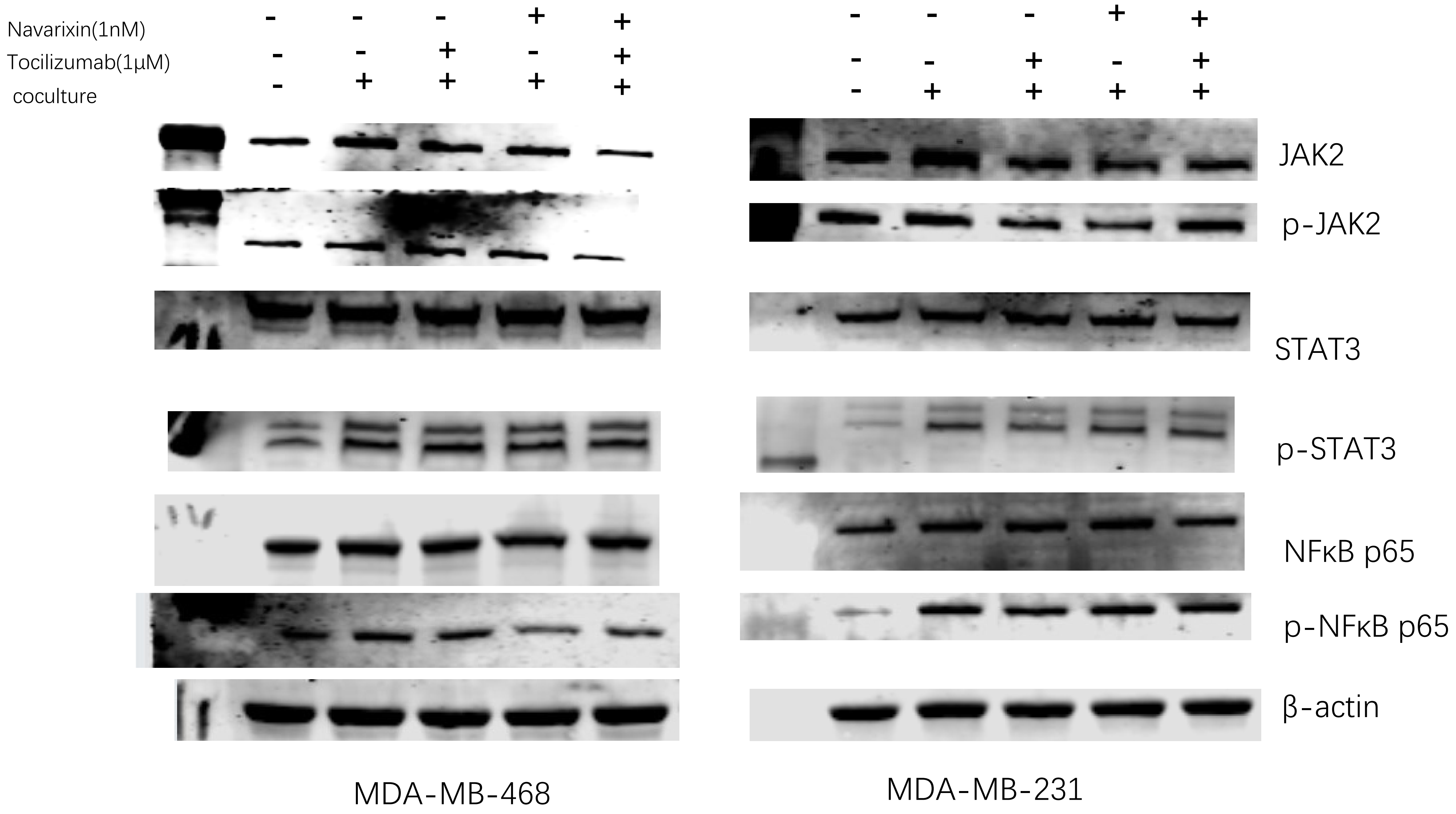
Navarixin(1nM)
Tocilizumab(1μM)
coculture
- - - + +
- - + - +
- + + + +
JAK2
p-JAK2
STAT3
p-STAT3
NFκB p65
p-NFκB p65
β-actin
MDA-MB-468
MDA-MB-231

Figure 4F

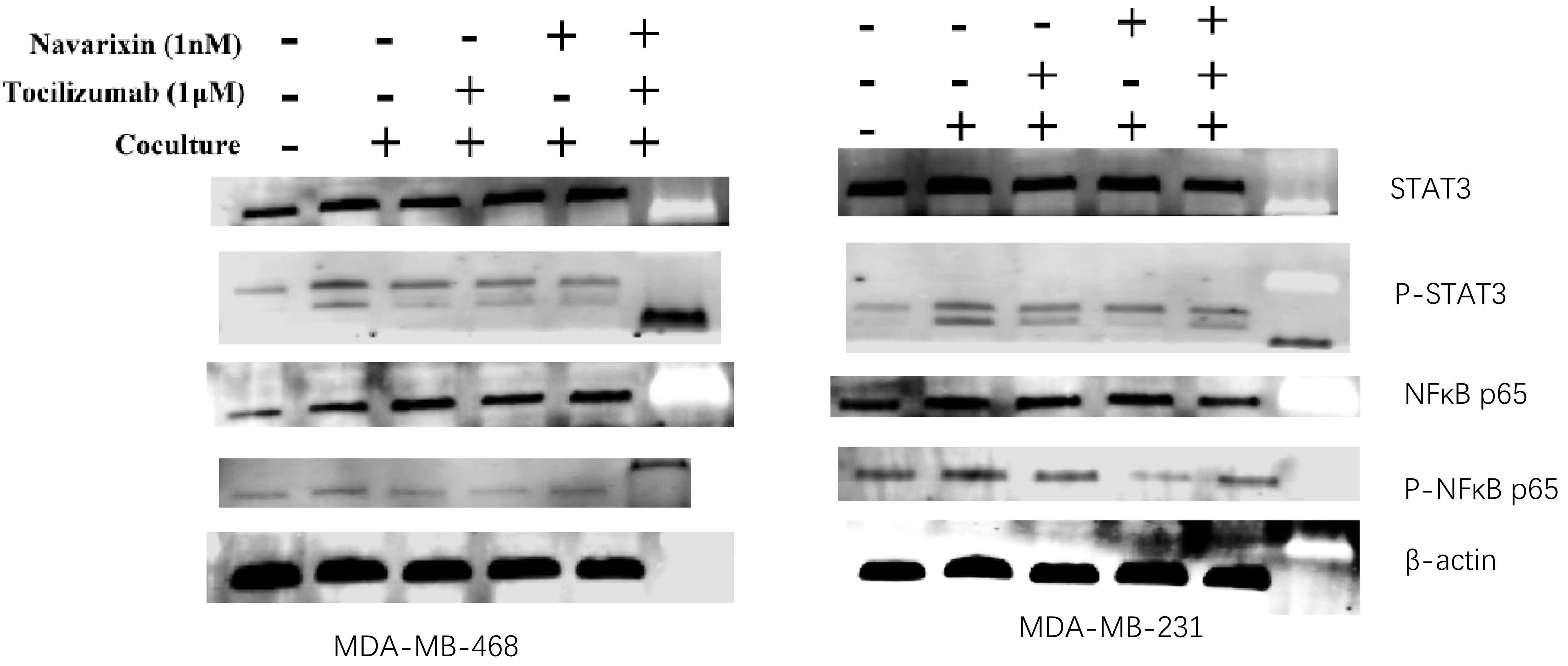

Navarixin (1nM) - - - + +
Tocilizumab (1μM) - - + - +
Coculture - + + + +
- - - + +
- - + - +
- + + + +
STAT3
P-STAT3
NFκB p65
P-NFκB p65
β-actin
MDA-MB-468
MDA-MB-231

Figure 5C

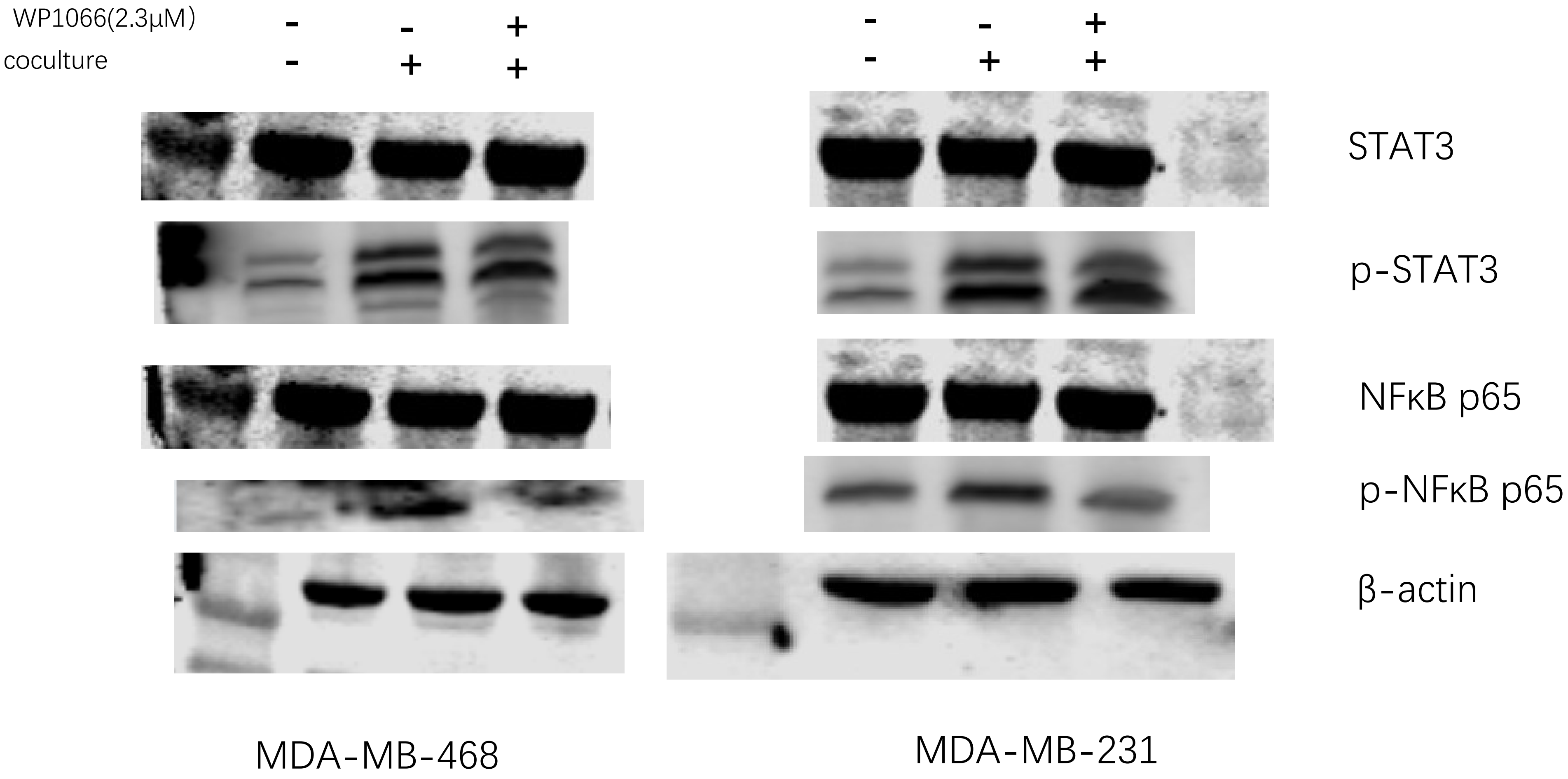

WP1066(2.3μM)
coculture
- - +
- + +
- - +
- + +
STAT3
p-STAT3
NFκB p65
p-NFκB p65
β-actin
MDA-MB-468
MDA-MB-231

Figure 5H

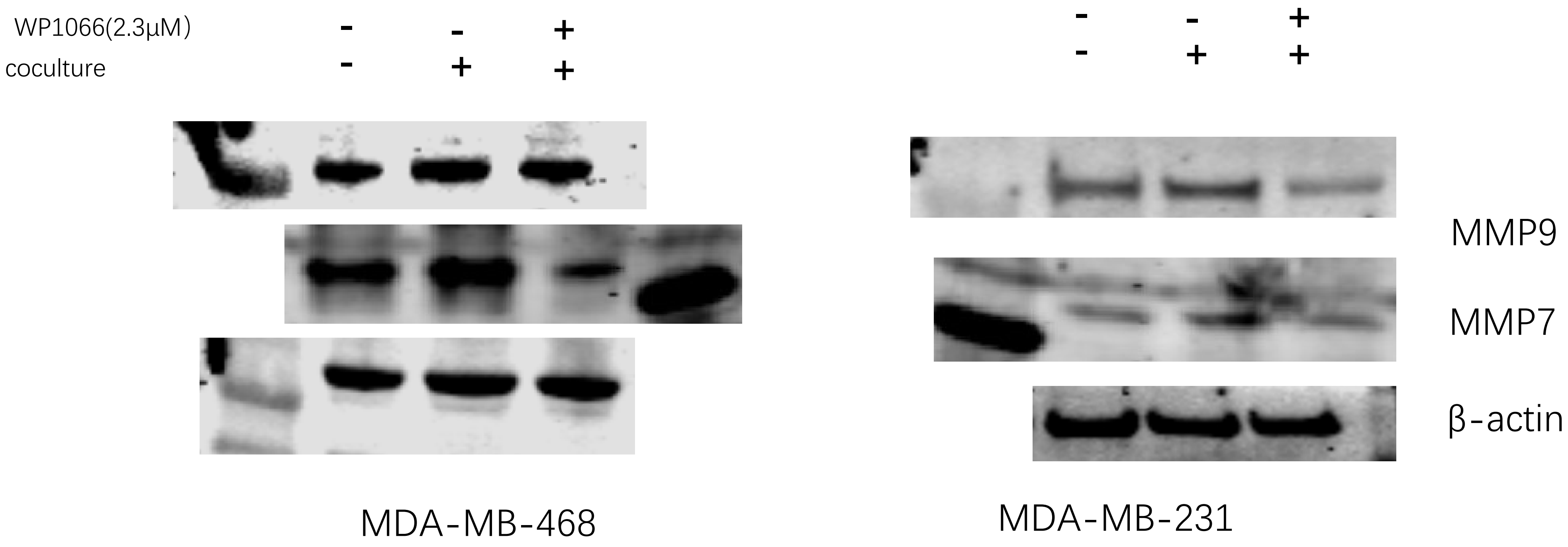

Figure 5K

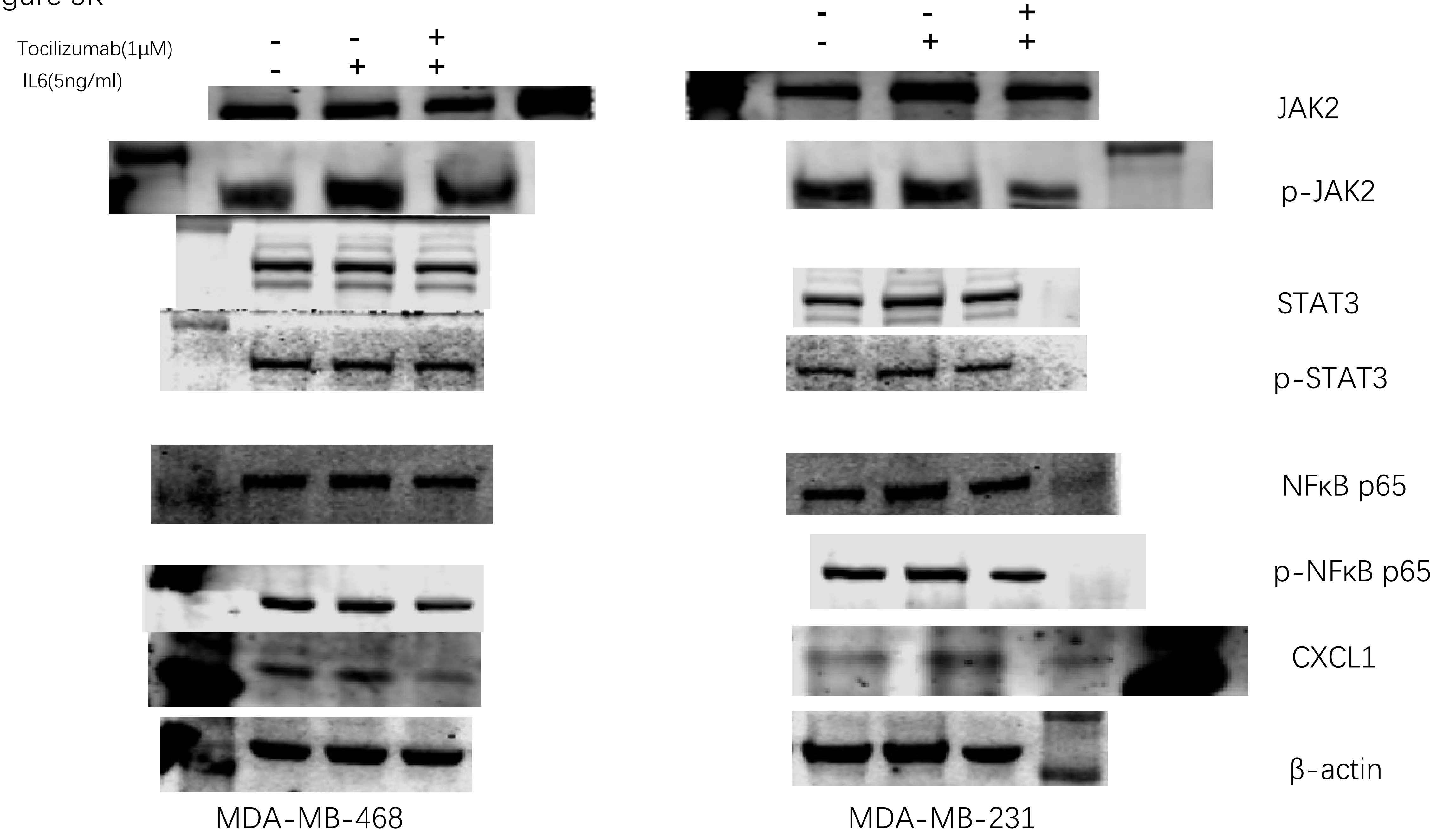

Tocilizumab(1μM)
IL6(5ng/ml)
- - +
- + +
- - +
- + +
JAK2
p-JAK2
STAT3
p-STAT3
NFκB p65
p-NFκB p65
CXCL1
β-actin
MDA-MB-468
MDA-MB-231

Figure 6G

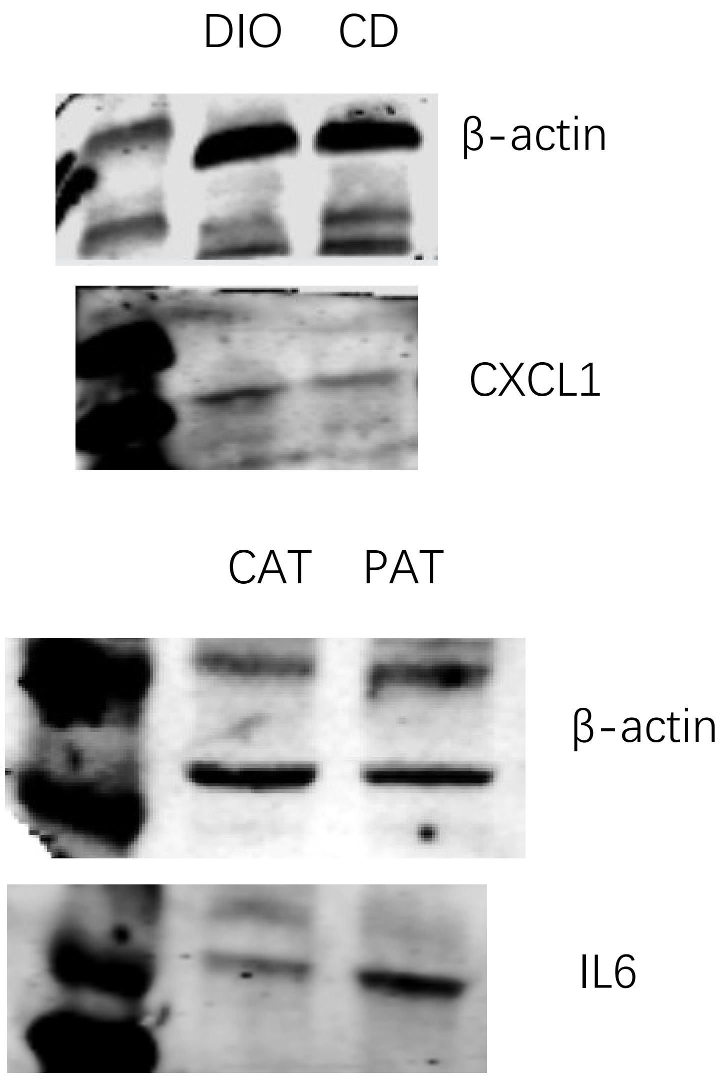

Figure 6H

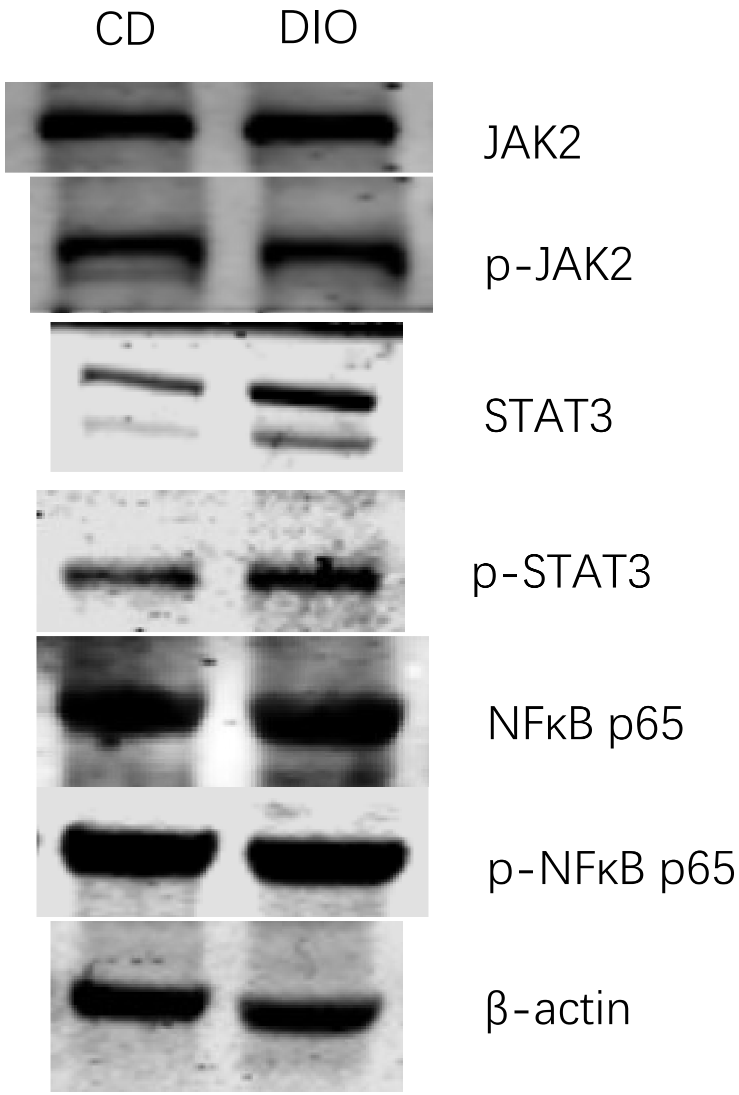

Figure 6I

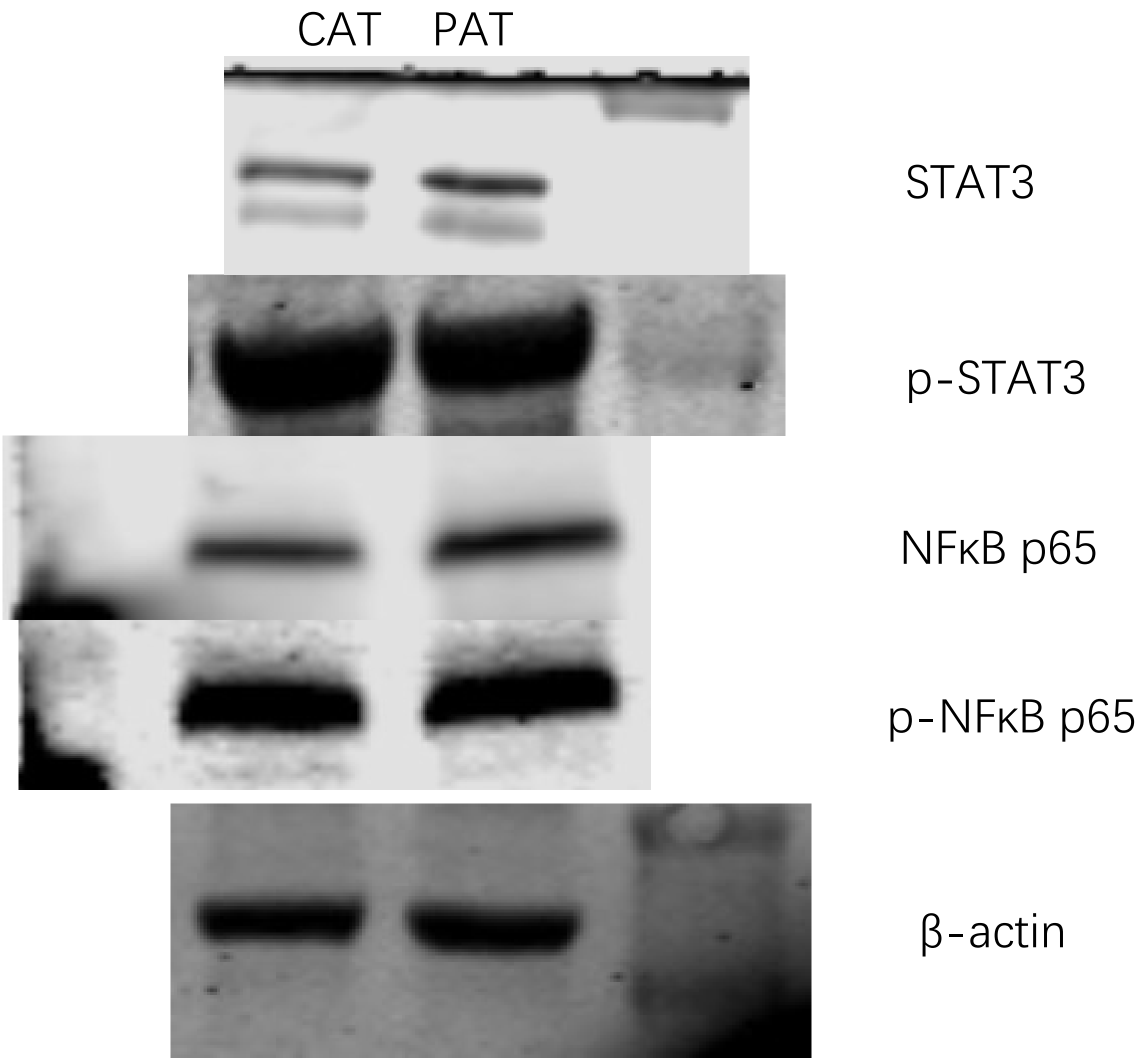

Figure 6J

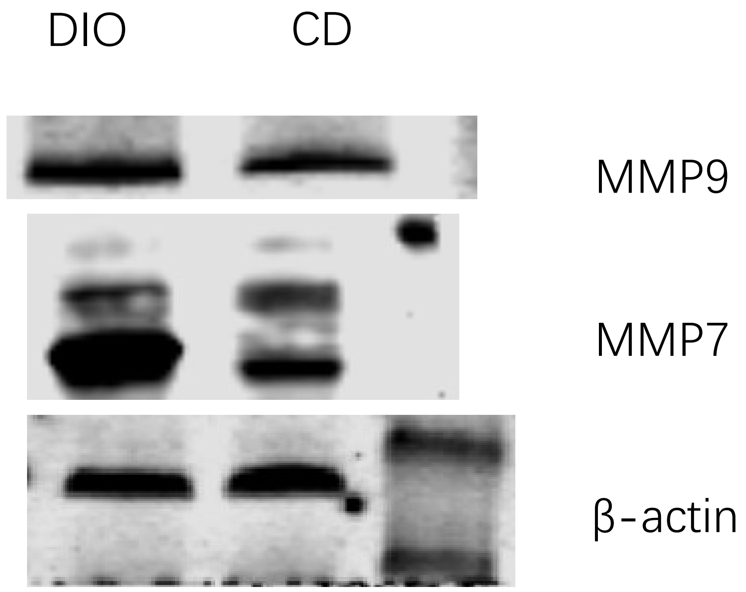

Supplement: Supplementary file 1 — Original Data [file 41420_2025_2713_MOESM1_ESM.pdf]
