# Supplementary material for: Adipocyte-derived IL6 and triple-negative breast cancer cell-derived CXCL1 co-activate STAT3/NF-κB pathway to mediate the crosstalk between adipocytes and triple-negative breast cancer cells
Source: Cell Death Discov. 2025 Aug 21;11:395. doi: 10.1038/s41420-025-02713-4 (PMC12370983; doi:10.1038/s41420-025-02713-4)
Supplement: Supplementary file 2 — Supplementary Figure legends [file 41420_2025_2713_MOESM2_ESM.pdf]

Supplementary Figure legends

Figure S1 Transcriptional sequence analysis of MDA-MB-468 cells co-cultured with or without 3T3-L1 cells.

MDA-MB-468 of TNBC cells were co-cultured with 3T3-L1 cells or cultured separately. After 3 days, the cells were collected for transcriptional sequencing. A, Differential gene volcano plot, yellow indicates differentially expressed up-regulated genes, blue represents differentially down-regulated genes; B, Differential gene heat map, red indicates differentially expressed up-regulated genes, blue represents differentially down-regulated genes. N=3 co-expressed MDA-MB-468 cells and n=3 single cultured MDA-MB-468 cells; C-D, GO terms bio-enrichment analysis and KEGG pathway analysis of differentially expressed genes. C, Bubble diagram of GO terminology biological enrichment analysis; D, Gene enrichment analysis circles of KEGG; D-N, Enrichment analysis of GSEA pathway of differentially expressed genes.

Figure S2 Transcriptional sequence analysis of hADSC cells co-cultured with or without MDA-MB-468 cells.

MDA-MB-468 of TNBC cells were co-cultured with 3T3-L1 cells or cultured separately. After 3 days, the cells were collected for transcriptional sequencing. A, Differential gene volcano plot, yellow indicates differentially expressed up-regulated genes, blue represents differentially down-regulated genes; B, Differential gene heat map, red indicates differentially expressed up-regulated genes, blue represents differentially down-regulated genes. N=3 co-expressed MDA-MB-468 cells and n=3 single cultured MDA-MB-468 cells; C-D, GO terms bio-enrichment analysis and KEGG pathway analysis of differentially expressed genes. C, Bubble diagram of GO terminology biological enrichment analysis; D, Gene enrichment analysis circles of KEGG; D-N, Enrichment analysis of GSEA pathway of differentially expressed genes.

Figure S3 Recombinant IL6 promotes TNBC cell migration and invasion.

MDA-MB-468 or MDA-MB-231 cells were treated with recombinant IL6 for 2 days, and the invasion experiment was carried out. A, Scratch healing experiment on MDA-MB-468 cells; B, Scratch healing experiment on MDA-MB-231 cells; C, Invasion experiment on MDA-MB-468 and MDA-MB-231cells.
