# Supplementary figures and images for: Adipocyte-derived IL6 and triple-negative breast cancer cell-derived CXCL1 co-activate STAT3/NF-κB pathway to mediate the crosstalk between adipocytes and triple-negative breast cancer cells
Source: Cell Death Discov. 2025 Aug 21;11:395. doi: 10.1038/s41420-025-02713-4 (PMC12370983; doi:10.1038/s41420-025-02713-4)

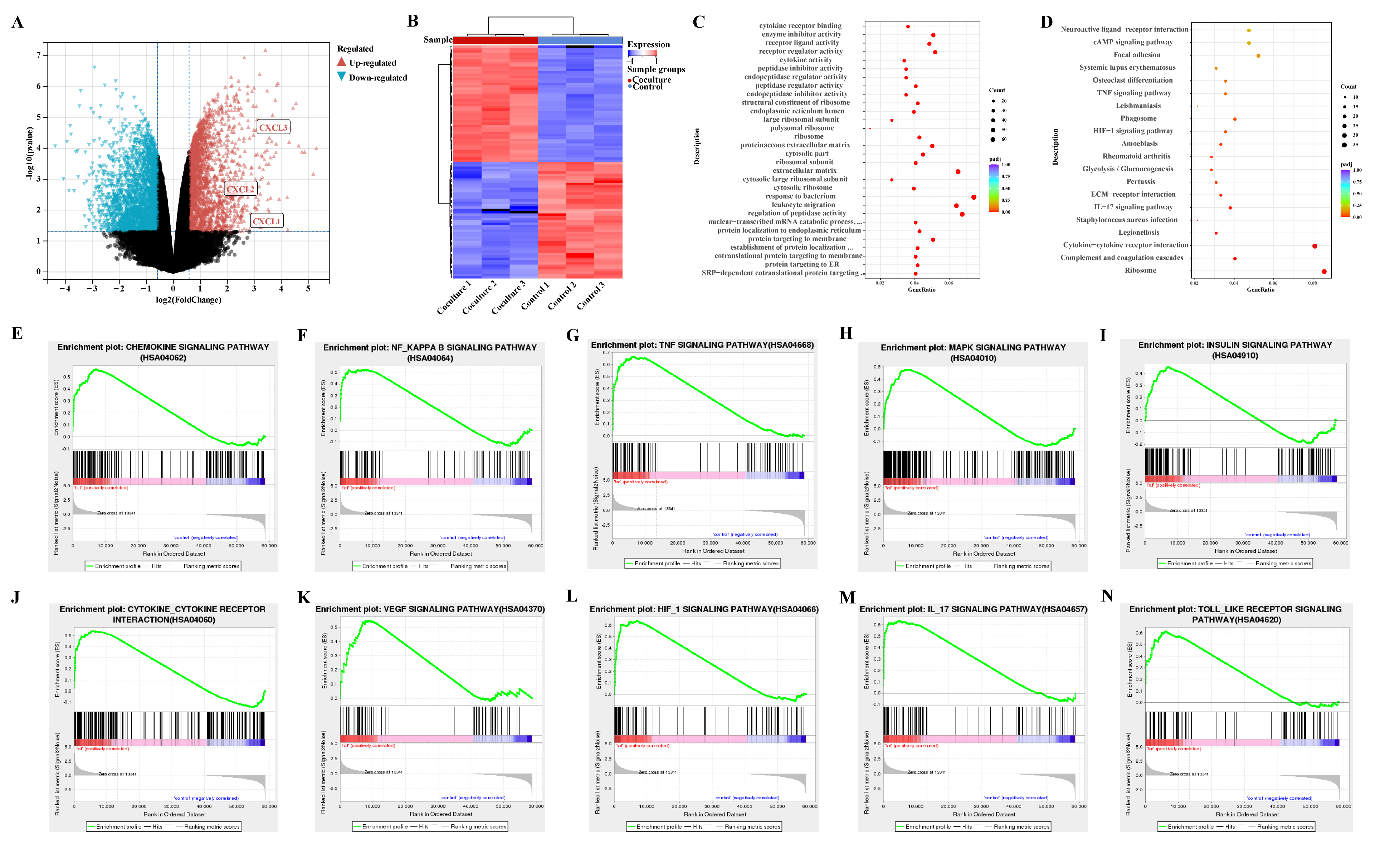

Supplement: Supplementary file 3 — Figure S1 [file 41420_2025_2713_MOESM3_ESM.tif]

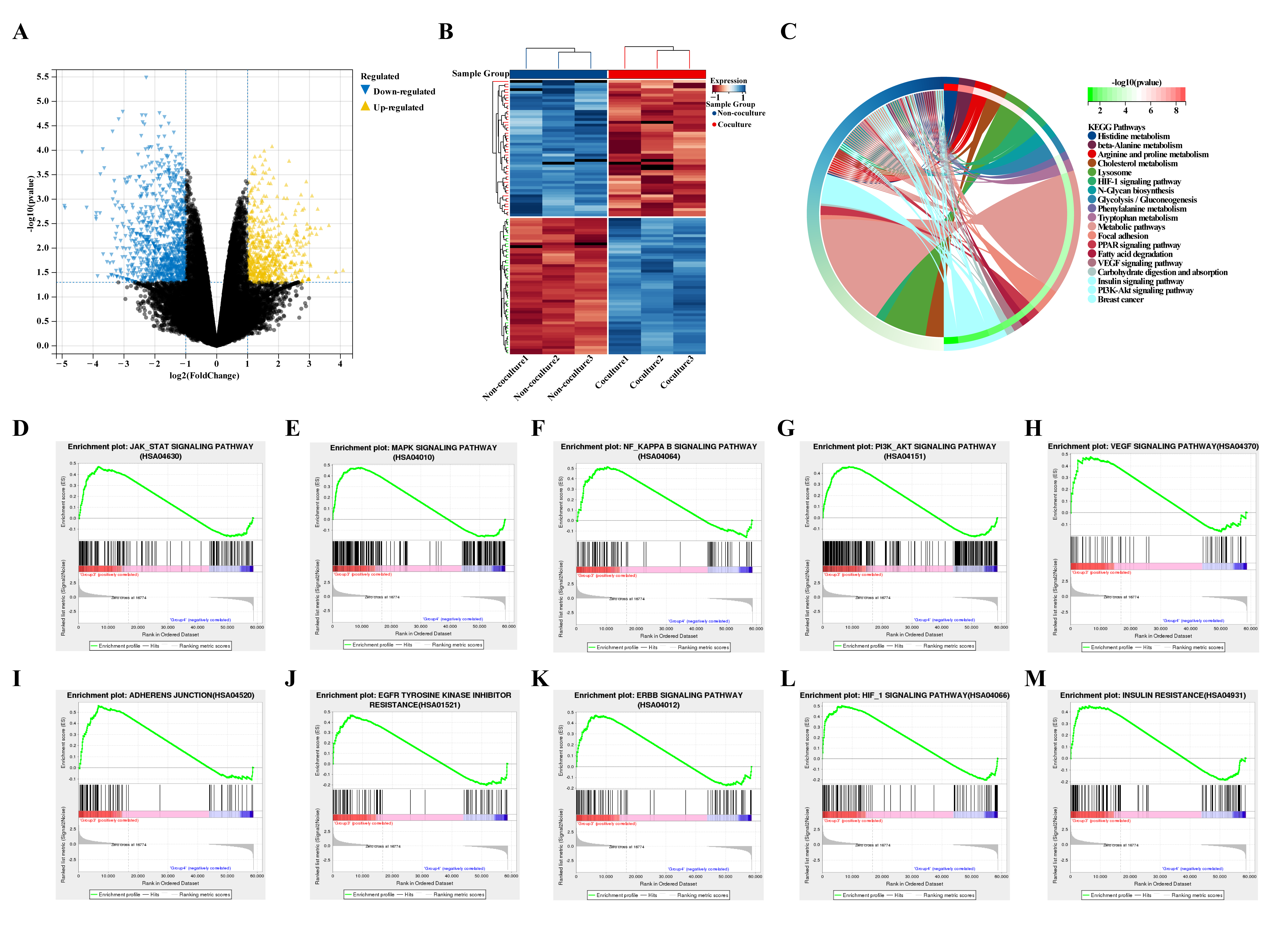

Supplement: Supplementary file 4 — Figure S2 [file 41420_2025_2713_MOESM4_ESM.tif]

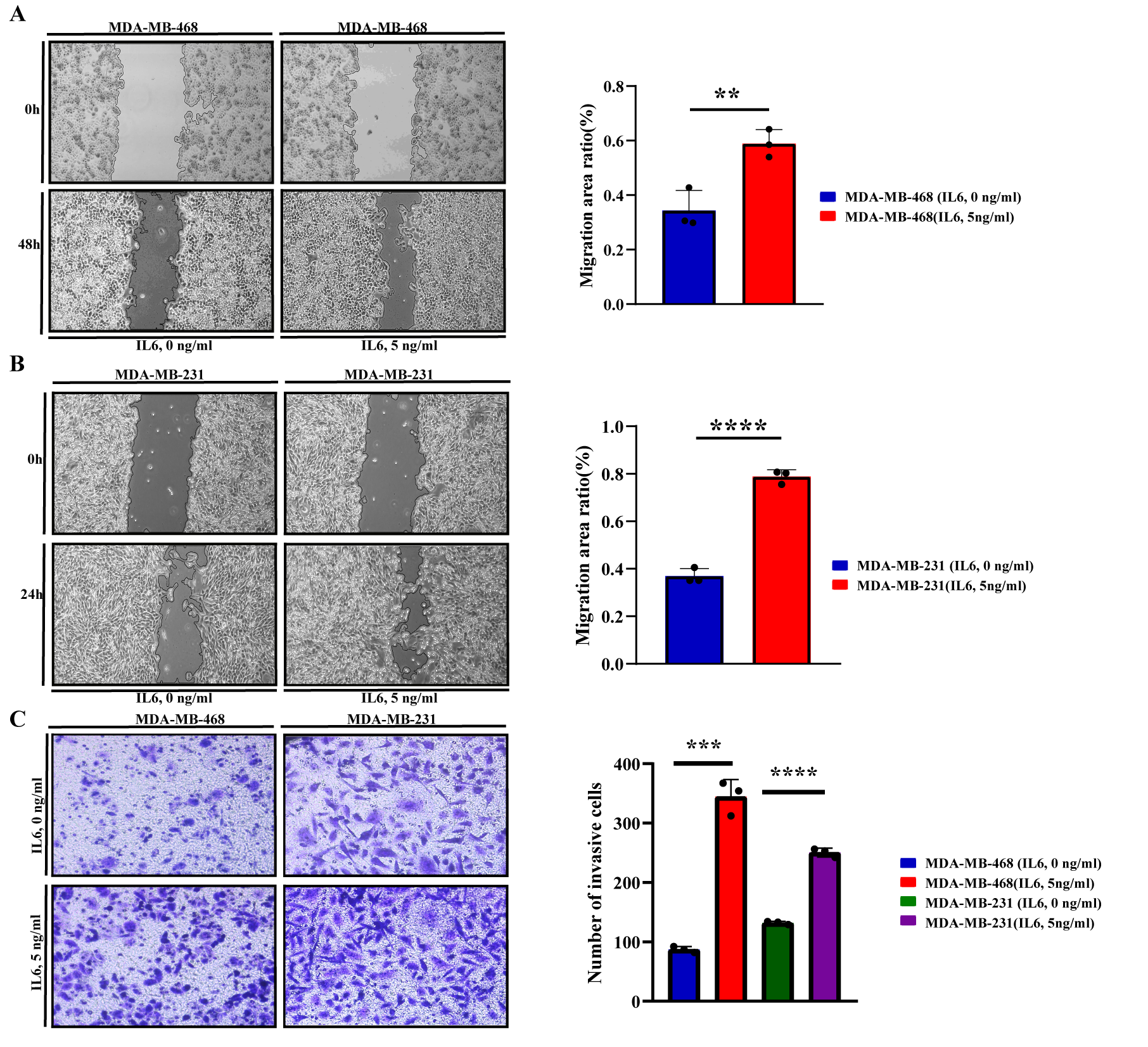

Supplement: Supplementary file 5 — Figure S3 [file 41420_2025_2713_MOESM5_ESM.tif]
