# Supplementary material for: Adipocyte-derived IL6 and triple-negative breast cancer cell-derived CXCL1 co-activate STAT3/NF-κB pathway to mediate the crosstalk between adipocytes and triple-negative breast cancer cells
Source: Cell Death Discov. 2025 Aug 21;11:395. doi: 10.1038/s41420-025-02713-4 (PMC12370983; doi:10.1038/s41420-025-02713-4)
Supplement: Supplementary file 6 — Table S1 [file 41420_2025_2713_MOESM6_ESM.docx]

**Table S1 Sequence of primers associated with Real-time PCR reaction**

| Gene symbol | Forward primer (5’ to 3’) | Reverse primer (5’ to 3’) |
| --- | --- | --- |
| GAPDH | GTCAGCCGCATCTTCTTT | CGCCCAATACGACCAAA |
| CXCL1 | AAGAACATCCAAAGTGTGAACG | CACTGTTCAGCATCTTTTCGAT |
| CXCL2 | AACCGAAGTCATAGCCACACTCAAG | TCCTCCTTCCTTCTGGTCAGTTGG |
| CXCL3 | AACCGAAGTCATAGCCACACTCAAG | CAGTTGGTGCTCCCCTTGTTCAG |
| CXCR2 | CCTGTCTTACTTTTCCGAAGGAC | TTGCTGTATTGTTGCCCATGT |
| IL6 | CACTGGTCTTTTGGAGTTTGAG | GGACTTTTGTACTCATCTGCAC |
| IL6R | CATGTGCGTCGCCAGTAGT | AGCTCAAACCGTAGTCTGTAGA |

Notes: GAPDH, glyceraldehyde-3-phosphate dehydrogenase; CXCL1, C-X-C motif chemokine ligand 1; CXCL2, C-X-C motif chemokine ligand 2; CXCL3, C-X-C motif chemokine ligand 3; CXCR2, C-X-C motif chemokine receptor 2; IL6, interleukin 6; IL6R, interleukin 6 receptor.
