# Supplementary material for: Adipocyte-derived IL6 and triple-negative breast cancer cell-derived CXCL1 co-activate STAT3/NF-κB pathway to mediate the crosstalk between adipocytes and triple-negative breast cancer cells
Source: Cell Death Discov. 2025 Aug 21;11:395. doi: 10.1038/s41420-025-02713-4 (PMC12370983; doi:10.1038/s41420-025-02713-4)
Supplement: Supplementary file 7 — Table S2 [file 41420_2025_2713_MOESM7_ESM.docx]

**Table S2 Antibody reagent.**

| Antibodies | Source | Identifier |
| --- | --- | --- |
| Anti-GAPDH antibody | Aksomics | KC-5G4 |
| Anti-β-actin antibody | Proteintech | 66009-1-Ig |
| Anti-CXCL1 antibody | Abcam | ab86436 |
| Anti-CXCL2 antibody | Thermo Fisher Scientific | DF12551 |
| Anti-CXCL3 antibody | LifeSpan Biosciences | LS-C807237 |
| IL6 antibody | Abcam | ab259341 |
| Recombinant Human IL-6 | Proteintech | 031916 |
| Anti-CXCR2 antibody | Proteintech | 20634-1-AP |
| Anti-IL6R antibody | Proteintech | 66855-1-lg |
| Anti-JAK2 antibody | Cell Signaling Technology | 3230 |
| Anti-JAK2 (phospho Y1007+Y1008) antibody [E132] | Abcam | ab32101 |
| Anti-STAT3 antibody | Abcam | ab9352 |
| Anti-STAT3 (phospho Y705) antibody [EP2147Y] | Abcam | ab76315 |
| Anti-NF-ΚB p65 (phospho) antibody [EP2294Y] | Abcam | ab76302 |
| Anti-NF-ΚB p65 antibody | PTMBIO | PTM-5591 |
| Anti-MMP7 antibody | Proteintech | 10374-2-AP |
| Anti-MMP9 antibody | Abcam | ab58803 |
| DyLight 800 AffiniPure Goat Anti-Mouse igG (H+L) | Earthox | E032810-01 |
| Goat Anti-Rabbit IgG H&L (Alexa Fluor® 488) | Abcam | ab150077 |
| Tocilizumab (Anti-Human IL6Rα) | MCE | HY-P9917 |
| Navarixin (SCH 527123) | MCE | HY-10198 |
| WP1066 (HY-15312) | MCE | HY-15312 |

Notes: GAPDH, glyceraldehyde-3-phosphate dehydrogenase; CXCL1, C-X-C motif chemokine ligand 1; CXCL2, C-X-C motif chemokine ligand 2; CXCL3, C-X-C motif chemokine ligand 3; CXCR2, C-X-C motif chemokine receptor 2; IL6, interleukin 6; IL6R, interleukin 6 receptor.
